# Harnessing in-situ tribo-plasma emission for energy harvesting

**DOI:** 10.1126/sciadv.aei0397

**Published:** 2026-09-09

**Authors:** Qixuan Zeng, Liming Tan, Fan Xu, Tingyu Wang, Ai Chen, Xue Wang

**Affiliations:** ^1^ College of Physics, Guizhou Province Key Laboratory for Photoelectrics Technology and Application, Guizhou University, Guiyang 550025, P. R. China.; ^2^ Department of Applied Physics, Chongqing University, Chongqing 400044, P. R. China.

## Abstract

Electrostatic discharge (ESD) in triboelectrification is conventionally dismissed as an uncontrolled energy dissipation process. Here, we introduce an approach to harness tribo-plasma generated by spontaneous ESD between sliding dielectric materials. Leveraging the intrinsic triboelectric field, this mechanism drives in situ plasma generation and guides its directional emission, enabling direct electrical power output via simple wire electrodes. The resulting current can be configured as either quasi-square-wave alternating current or direct current, depending on the motion profile. Notably, the device exhibits exceptional constant-current performance, sustaining output with a minimal decay of 0.32 microampere at a load resistance of 500 megohms. The universality of this mechanism is validated across diverse materials, including polymers, fabrics, semiconductors, and metals. Beyond energy harvesting, the tribo-plasma generated by mechanical motion also shows proof-of-concept potential for surface modification, food preservation, and ion implantation. These findings establish a potential route for converting mechanical energy into electrical output and provide a foundation for power-source–free plasma technologies.

## INTRODUCTION

Tribological phenomena are ubiquitous at interfaces in both natural and engineered systems involving relative motion and inherently couple multiple physical processes, including wear, heat generation, material transfer, and charge transfer (*1*–*3*). Among these processes, charge transfer induced by tribological contact, namely, triboelectrification, has long been recognized as a fundamental electrical effect (*4*–*6*). Particular attention has been paid to this phenomenon in the field of triboelectric nanogenerators (TENGs), where triboelectrification serves as one of the key mechanisms enabling the conversion of mechanical energy into electricity (*7*, *8*). However, triboelectrification often leads to local charge accumulation at the interface, which can generate intense electrostatic fields (*9*, *10*). In sliding contact, once the field strength exceeds the dielectric breakdown threshold of the surrounding medium, electrostatic discharge (ESD) can be triggered (*11*–*13*), resulting in the rapid release of stored electrostatic energy. Although the ESD process exhibits a high instantaneous energy density, its stochastic and transient nature has severely hindered the efficient capture and practical utilization of this energy (*11*, *14*).

Against this background, direct-current TENGs (dc-TENGs) have been developed as an effective strategy for harvesting ESD energy (*15*, *16*). By introducing an artificial metal-dielectric microgap, these devices can localize and exploit ESD, thereby enabling direct energy conversion and dc output (*17*, *18*). Despite the recent progress enabled by this strategy, most existing modeling frameworks still regard ESD as a transient conductive or ionization pathway that facilitates the rapid transfer or direct collection of triboelectric charges (*19*, *20*). Accordingly, the design of dc-TENGs typically relies on metal-dielectric microgaps and sharp electrode architectures to deliberately intensify local electric fields and initiate ESD. In contrast to this engineered discharge mode, ESD can arise spontaneously during sliding contact between dielectric materials (*21*, *22*), manifesting as spatially distributed events at and around the sliding contact interface. These ESD events result from electron-avalanche ionization, thereby generating tribo-plasma containing various charged particles, including electrons and both positive and negative ions (*23*–*25*). This process is often accompanied by interfacial phenomena such as triboemission and triboluminescence (*26*–*28*). From an energy conversion perspective, these charged species endow tribo-plasma with substantial yet largely untapped energy potential. However, its intrinsic quasi-neutrality makes the efficient extraction of this energy and its direct conversion into usable electrical output fundamentally difficult (*29*). Therefore, a comprehensive investigation of tribo-plasma generated in purely dielectric sliding systems may contribute to a deeper understanding of triboelectrification and provide further insights into energy conversion.

In this study, we present an approach for triboelectric energy conversion based on the utilization of in situ generated tribo-plasma. The key to this approach lies in the intrinsic coupling between plasma generation and its directional emission. The sliding motion can induce spontaneous plasma formation, while the local triboelectric field functions as a natural charge separator that governs the emission direction. To elucidate the underlying physical mechanisms, we systematically investigate the process of tribo-plasma generation, map the spatial distribution of the governing triboelectric field, and develop a physical model describing electric field–driven directional plasma emission. According to this model, the sliding system can deliver either a quasi-square-wave ac or a dc output, depending on the mechanical motion mode. Furthermore, the mechanism is applicable to a wide range of material systems, including polymers, fabrics, semiconductors, and metals. The energy conversion approach exhibits excellent constant-current performance, with a current decay of only 0.32 μA (8.3%) at a high load resistance of 500 MΩ. In addition, we demonstrate that the in situ plasma, driven by mechanical excitation, can be directly used in applications such as surface modification, food preservation, and ion implantation. These findings not only deepen the understanding of triboelectrification and plasma science but also provide a foundation for the development of energy conversion strategies and passive plasma technologies.

## RESULTS

### In situ tribo-plasma emission and the conceptual framework of the bc-TENG

**Fig. 1.**
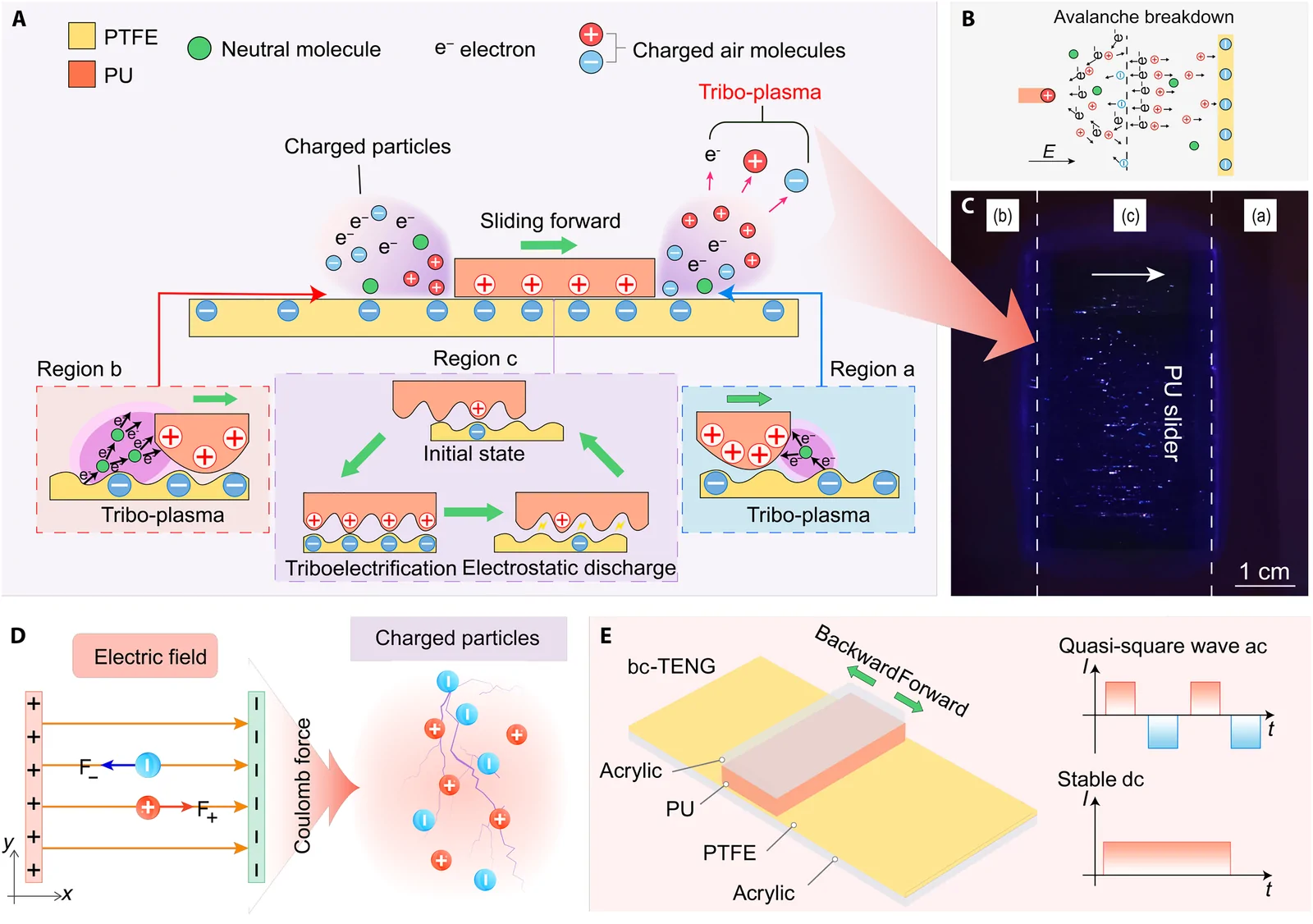
Tribo-plasma generation and the conceptual framework of the bc-TENG. (**A**) Conceptual schematic of tribo-plasma formation in the sliding system, with insets illustrating the proposed discharge behavior in different regions. (**B**) Schematic diagram of the air avalanche breakdown mechanism. (**C**) Photograph of the sliding interface in a darkened environment, visualizing the discharge phenomena across various regions. (**D**) Schematic illustrating the regulation of charged particles by an electric field via the Coulomb force. (**E**) Conceptual design of the bc-TENG and its bicharacteristic current output capability.

Beyond the extensively studied phenomenon of triboelectrification, which leads to the accumulation of electrostatic charges on surfaces during sliding contact, the present study reports a directional emission behavior of tribo-plasma. Unlike conventional plasmas that often require external energy sources or complex instrumentation, tribo-plasma offers distinct advantages. It is intrinsically generated at room temperature by the intense, localized electric fields arising from triboelectrification, rendering it self-sustained and easily scalable for portable applications without the need for high-voltage supplies or elaborate setups (*23*, *26*). Here, we mainly demonstrate the directional emission of tribo-plasma within a polymer-based triboelectric system, induced by simple mechanical sliding. As a prototypical demonstration, a block-on-flat sliding configuration consisting of a polyurethane (PU) slider and a polytetrafluoroethylene (PTFE) stator was used. These materials were selected for their relatively large difference in electronegativity and favorable contact property, which result in a pronounced triboelectric effect. This configuration generates a continuous tribo-plasma at both the leading and trailing edges of the PU slider as it moves across the PTFE film (Fig. 1A). The resulting tribo-plasma consists of positive ions, negative ions, and electrons, which subsequently disperse into the surrounding environment. The underlying mechanism involves the excitation and ionization of ambient air molecules by the intense local electric fields generated at the sliding interface. When the field strength exceeds the air breakdown threshold, an electron avalanche is initiated. The triboelectric field accelerates seed electrons, which subsequently collide with and ionize ambient air molecules. This process of impact ionization generates a cascade of secondary electrons and ions (e^−^ + M → e^−^ + M^+^ + e^−^), culminating in the formation of plasma, as schematically depicted in Fig. 1B. Specifically, upon repetitive sliding cycles, the electronegativity difference between PU and PTFE leads to the accumulation of positive charges on the PU surface and negative charges on the PTFE surface. A notable characteristic of this system is the nonsaturating nature of the surface charge density, maintained by a dynamic cycle of triboelectrification and discharge during sliding contact (fig. S1A and note S1). To elucidate the spatial distribution and generation mechanism of the tribo-plasma, the system is segmented into three distinct regions along the sliding direction: the leading edge (a), the trailing edge (b), and the central contact interface (c), as illustrated in the insets of Fig. 1A. At the leading edge (a), as the PU slider advances and establishes fresh contact with the PTFE surface, positive charges accumulate at the front of the slider. These positive charges, in conjunction with residual negative charges on the PTFE surface ahead of the contact zone, establish a strong localized electric field [Fig. 1A, inset region (a)]. When the magnitude of this field surpasses the dielectric breakdown strength of air, gas ionization is triggered, leading to the formation of a plasma emission source. A similar process occurs at the trailing edge (b), where the positively charged trailing end of the PU interacts with the freshly exposed, negatively charged PTFE surface, again forming a strong local field and initiating air ionization through dynamic discharge [Fig. 1A, inset region (b)]. Dark-field optical photography was used to visualize the spatial profile of the discharge. As shown in Fig. 1C, a distinct bluish-purple glow is observed, which is consistent with the well-known phenomenon of triboluminescence (*30*). Crucially, this spatial distribution supports our model, revealing that discharges are concentrated at the leading and trailing edges [region (a) and region (b)]. In the central contact region (c), the porous microstructure of the PU foam creates numerous microscale air gaps at the sliding interface [Fig. 1A, inset region (c)]. A comparative experiment employing a smooth PU block devoid of surface microstructures was performed to verify the function of these features. This comparison confirmed that the porous architecture is instrumental in enabling discharge initiation and charge transfer within the contact region (fig. S2). Specifically, within these tiny spaces, opposing triboelectric charges generate exceptionally strong electric fields that often exceed the dielectric breakdown strength of air (≥3 kV/mm). Finite element simulations (COMSOL Multiphysics; fig. S1B) corroborate these field strengths. These intense fields induce continuous micro-discharges within the gaps. Discrete luminescent spots observed in the optical photograph (Fig. 1C) provide direct visual evidence of these internal microdischarging events, demonstrating that triboelectrification and discharge proceed concurrently across the contact area. These findings establish that tribo-plasma emission is driven by the dynamic synergistic interaction between triboelectrification and ESD. The primary emission sources are located at the leading and trailing edges, where intense and localized electric fields continuously ionize the ambient air. This inherent connection between the intrinsic triboelectric field and plasma formation paves the way for a self-contained energy system, presenting the compelling prospect of leveraging the same electric field that induces the plasma to subsequently separate its constituent charged particles for direct electrical power generation. The effective separation of charged particles is a foundational principle of plasma-based energy conversion technologies. In a magnetohydrodynamic generator, the Lorentz force generated by an external magnetic field drives the separation of charged particles in ionized gas. Analogously, charged particles can also be separated under the Coulomb force of an electric field, as illustrated in Fig. 1D. This provides a conceptual basis for electric field–driven charge separation in the present system. Based on this mechanism, a bicharacteristic current TENG (bc-TENG) was constructed using a PU slider and a PTFE-covered stator (Fig. 1E). The electrical power is extracted by copper wire electrodes, in either insulated or exposed configurations, which are positioned along the sides of the PU slider (fig. S3 and movie S1). A defining characteristic of this prototype is that it can deliver two distinct output modes from the same physical mechanism. During reciprocating sliding, it produces a quasi-square-wave ac, whereas continuous unidirectional motion yields a stable dc (the inset in Fig. 1E). This dual-mode capability arises from the intrinsic triboelectric field, which not only generates tribo-plasma but also directs its constituent charged particles. The interaction between plasma formation and charge separation therefore determines the characteristics of the electrical output.

### Working principle of the bc-TENG and tribo-plasma emission characteristics

**Fig. 2.**
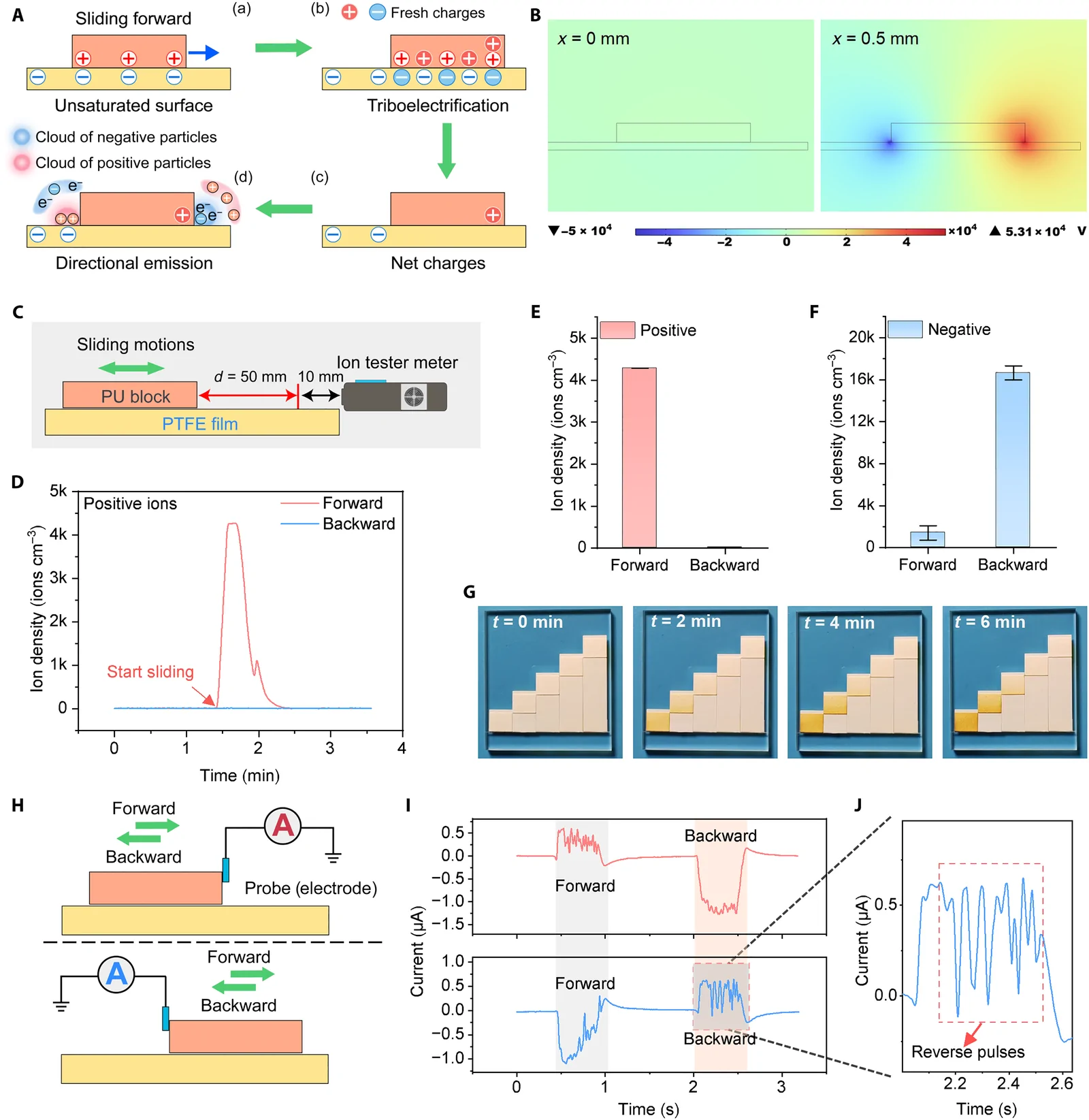
Working mechanism of the bc-TENG and characterization of its tribo-plasma emission. (**A**) Schematic diagram of the bc-TENG’s working principle. (**B**) Simulated electric potential distribution at the initial position (*x* = 0 mm) and after a forward displacement of 0.5 mm (*x* = 0.5 mm). (**C**) Schematic of the experimental setup for measuring ion concentration. (**D**) Temporal evolution of positive ion concentration within a single reciprocating cycle. (**E** and **F**) Average concentrations of (E) positive and (F) negative ions over three reciprocating cycles. (**G**) Photographs of ozone test strips demonstrating colorimetric changes indicative of ozone generation. (**H**) Schematic of the probe measurement configuration, depicting the probe positioned on the right (top schematic) and left (bottom schematic) sides of the PU slider. (**I**) Current signals generated by the tribo-plasma, measured using a metal probe at the slider’s right and left edges. (**J**) Enlarged view of the current signal from the trailing edge during the slider’s backward stroke.

To elucidate the governing principle of the triboelectric field, we investigated the charge distribution during sliding contact, revealing a dynamically evolving dipole-like triboelectric field at the interface. The electric field pattern arises because ESD inherently lags behind the triboelectrification process. Specifically, in the absence of ESD, as the PU slider advances across the PTFE surface, its leading edge, where new contact continuously occurs, generates net positive charges. Concurrently, the trailing edge, where separation has just occurred, exposes the net negative charges on the PTFE surface [Fig. 2A, stages (a) to (c)]. This charge arrangement establishes a dipole-like electric field aligned with the sliding direction, with a positively charged front and a negatively charged rear. The validity of this dipole model is corroborated by finite element analysis. A 0.5-mm forward displacement produces a dipolar potential profile, positive at the front and negative at the rear, consistent with the predicted electric field pattern (Fig. 2B and fig. S4). These oppositely oriented electric fields provide an ideal driving force for spatially separating the charged particles in the above-mentioned tribo-plasma. Via Coulomb interactions, the positive field at the leading edge repels positive ions, while the negative field at the trailing edge repels negative ions [Fig. 2A, stage (d)]. Therefore, under continuous motion, the distinct electric field patterns at the leading and trailing edges of the slider play a dual role. They continuously generate tribo-plasma via ESD while simultaneously separating the resulting positive and negative ions. This mechanism drives the directional transport of charged particles, expelling positive ions from one end and negative ions from the other. Notably, when the slider reverses direction, both the electric field and the potential distribution reverse (fig. S5), causing a corresponding reversal in the polarity of the emitted ions.

An ion concentration measurement platform was constructed to experimentally validate the directional emission model. The inlet of the ion counter was positioned horizontally above the PTFE surface and oriented towards the slider (Fig. 2C and fig. S6), maintaining a separation distance of 10 mm at the rightmost end point of its reciprocating motion. Before initiating slider motion, the ambient ion concentration was confirmed to be low and stable, establishing a clear baseline for the measurement (fig. S7). However, upon the onset of the slider’s forward motion, a sharp and transient surge in positive ion concentration was detected. This spike momentarily saturated the detector’s measurement range before gradually returning to the baseline through diffusion and neutralization (Fig. 2D and movie S2). Conversely, no appreciable increase in the positive-ion concentration was observed during the slider’s reverse motion. The statistical analysis of repeated measurements reinforces this directional emission model. Positive ions are emitted exclusively during forward motion, whereas negative ions are predominantly detected during reverse motion (Fig. 2, E and F). These results provide compelling and direct evidence in support of the directional tribo-plasma emission mechanism. Furthermore, to confirm the presence of plasma and assess its spatial extent, we used an indirect chemical characterization method. Since air-discharge plasmas typically generate ozone, we utilized ozone-sensitive test strips to map the spatial distribution of tribo-plasma. An array of five test strips was affixed to a substrate facing the sliding interface, positioned at a vertical separation of 15 mm and moved synchronously with the slider (Fig. 2G and fig. S8). The results reveal a discernible color change in proximity to the slider’s edges, extending over a distance of ∼3 cm. This finding chemically corroborates the gas discharge at the slider’s edges and demonstrates that the plasma emission possesses an effective range on the centimeter scale.

To further probe the real-time electrical signature of tribo-plasma, the ion-induced currents were dynamically recorded using metal probes (Fig. 2H). As illustrated in Fig. 2I, the measured currents exhibit a clear correlation with the direction of motion. Specifically, during forward motion, the right probe registers a positive current peak, while the left probe detects a negative one. Upon reversal of motion, the polarities of these current peaks correspondingly invert. Notably, the positive current peaks are often followed by reverse pulses (Fig. 2J). This observation is attributed to the partial collection of highly mobile electrons from the tribo-plasma, a finding consistent with the small quantities of negative ions detected by the ion concentration meter during forward motion (Fig. 2F). Subsequently, to elucidate the thermodynamic nature of the generated plasma, we conducted real-time infrared thermal imaging of the sliding system (fig. S9A). Although the temperatures of the PU slider and the PTFE surface increased marginally by ∼1.1° and ∼2.2°C (fig. S9B), respectively, during 10 min of continuous motion, the key plasma emission regions, namely, the gas zones adjacent to the slider’s leading and trailing edges, remained at ambient temperature throughout the entire motion cycle. This thermal stability is further corroborated by the consistent and steady output current waveform (fig. S9C). These findings confirm that the tribo-plasma is nonthermal, a key characteristic of cold plasma. This nonthermal, power-source-free nature unlocks its potential for a variety of practical applications where thermal plasma sources are unsuitable, including surface modification, food preservation, and ion implantation.

### ac output of the sliding-mode bc-TENG

**Fig. 3.**
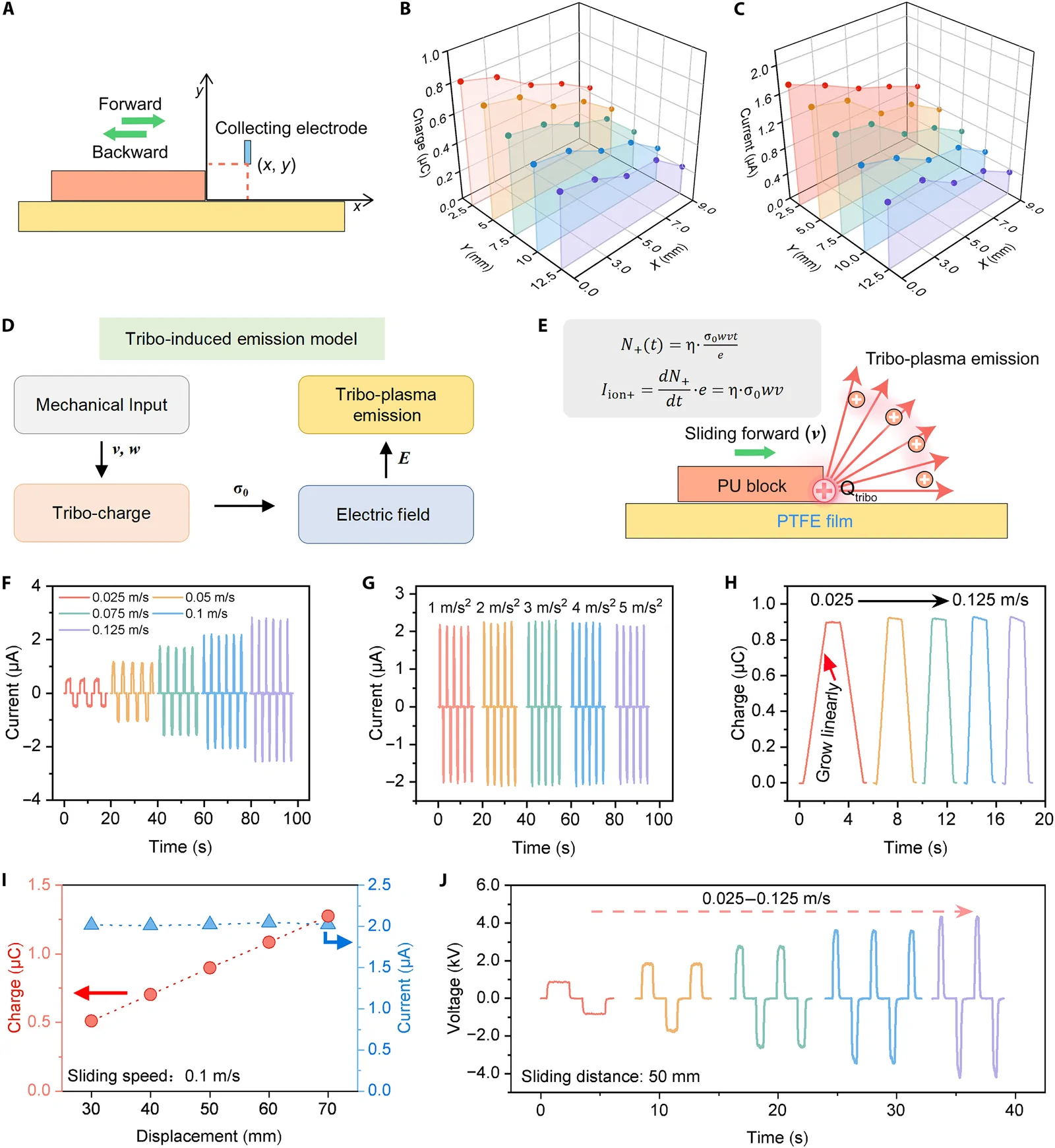
Electrical output characteristics of the sliding-mode bc-TENG and the underlying tribo-plasma emission model. (**A**) Schematic of the experimental setup using a movable electrode pair to map the spatial distribution of the electrical output. (**B** and **C**) Spatial maps of the output current (B) and transferred charge (C) measured at discrete positions within a defined area (*x*: 0 to 9 mm, Δ*x* = 3 mm; *y*: 2.5 to12.5 mm, Δ*y* = 2.5 mm). (**D**) Schematic flowchart illustrating the proposed tribo-plasma emission model, where the mechanically induced triboelectric field drives directional plasma emission. (**E**) Schematic of the tribo-plasma emission model, showing key influencing parameters: triboelectric charge density (σ_0_), sliding velocity (*v*), and slider width (*w*, the dimension perpendicular to the sliding direction). (**F**) Output current waveforms at varying accelerations (1 to 5 m/s^2^), demonstrating the output’s independence from acceleration. (**G** and **H**) Dependence of the output current (G) and transferred charge (H) on sliding velocity (0.025 to 0.125 m/s). (**I**) Output current and transferred charge as functions of sliding displacement (30 to 70 mm). (**J**) Output voltage waveforms measured across a 2-GΩ external load at various sliding velocities.

Leveraging the directional characteristics of tribo-plasma emission, a bc-TENG was developed to realize and examine this energy conversion concept. The device can be configured in either a sliding or a rotating mode to harvest linear or rotational mechanical energy, respectively. In the sliding mode, two Cu foils were mounted on the leading and trailing edges of the PU slider, serving as electrodes for electrical signal acquisition (fig. S10). To characterize the effective emission range of the tribo-plasma–based conversion mechanism, we measured output signals using a movable electrode pair at various spatial locations (horizontal distance: *x* mm, vertical distance: *y* mm) (Fig. 3A). The results reveal that stable electrical signals [transferred charge (*Q*) and short-circuit current (*I*_SC_)] were consistently detected over centimeter-scale distances (Fig. 3, B and C). Both the current and transferred charge decreased approximately linearly with increasing collection distance. Even with both the horizontal and vertical separations exceeding 9 mm, the device still achieves an *I*_SC_ of 0.4 μA and a *Q* of 200 nC (fig. S11). At extended collection distances, an asymmetry was observed between the positive and negative current and transferred-charge waveforms, which may arise from differences in the migration abilities of positively and negatively charged species in air. To elucidate the physical origin of the electrical signals and distinguish tribo-plasma emission from conventional air breakdown and electrostatic induction, a comparative experiment was conducted using exposed and insulation-covered electrode configurations (fig. S12A). The results demonstrate that both electrode configurations generate clear current signals. At a vertical distance of 2.5 mm (horizontal distance: 0 mm), the maximum currents reach 1.8 μA (exposed) and 1.2 μA (covered) (fig. S12B), with qualitatively similar *Q* profiles (fig. S12C). Crucially, even when the direct breakdown pathway is completely obstructed, the insulation-covered electrode still registers an *I*_SC_ of 0.3 μA at a vertical distance of 12.5 mm. The bc-TENG output was further compared with the electrostatic-induction response of a conventional contact separation mode TENG (CS-TENG) (fig. S13). The CS-TENG exhibited short-lived current transients and a rapid variation in electrical output over a small separation range. This finding provides compelling evidence that the primary energy conversion pathway is tribo-plasma emission followed by ion collection, whereas direct discharge and electrostatic induction may serve as secondary contributors. This conclusion is further corroborated by an experiment using a multiple-electrode array (fig. S14 and note S2).

To quantitatively describe the tribo-plasma–based energy conversion process, we propose an ion emission model driven by the triboelectric field (Fig. 3, D and E). Taking the leading edge of the PU slider as a representative case, the net positive triboelectric charge (*Q*_*tribo*_) is proportional to the contact area. Consequently, the total number of emitted positive ions (*N*_*+*_) and the resulting current (*I*_*ion+*_) can be expressed as (note S3)

$$N_{+}\left(t\right)=\eta \cdot \frac{Q_{\text{tribo}}\left(t\right)}{e}=\eta \cdot \frac{\sigma_{0}\mathrm{wvt}}{e}$$ (1)

$$I_{\text{ion}+}=\frac{dN_{+}}{\mathrm{dt}}\cdot e=\eta \cdot \sigma_{0}\mathrm{wv}$$ (2)

where η is the ion emission efficiency, *w* is the slider width (defined as the edge length perpendicular to the direction of motion), *v* is the sliding velocity, *t* is the movement time, σ_0_ is the surface net charge density, and *e* is the elementary charge.

To rigorously validate this model, we systematically investigated the influence of the slider’s kinematic parameters on the output performance. The experimental results align remarkably well with the model’s predictions. First, the output current amplitudes exhibit a direct linear dependence on sliding speed, reaching up to 3 μA at 0.125 m/s while remaining independent of acceleration (Fig. 3F and 3G). In contrast to the pulsed current characteristic of conventional ac-TENGs, the output waveform here exhibits a distinctive quasi-square-wave profile. Second, for a fixed sliding distance of 50 mm, the total transferred charge remains constant at ∼900 nC (Fig. 3H), irrespective of the sliding speed. Third, at a constant velocity, the transferred charge increases linearly with sliding distance, while the current amplitude remains unchanged (Fig. 3I). These observations provide a robust validation of our ion emission model (Fig. 3E). Furthermore, under a 2-GΩ external load, the device generates a voltage of up to 4.3 kV at a sliding speed of 0.125 m/s, confirming its outstanding energy output capability (Fig. 3J). Moreover, comparative analysis under identical operating conditions indicates that the bc-TENG markedly outperforms conventional contact electrification–based TENGs (fig. S15A). In particular, the bc-TENG achieves a 2.37-fold increase in contactless collected charge compared with the conventional device (fig. S15B). This substantial improvement is primarily ascribed to the avalanche breakdown mechanism, which promotes the formation of abundant tribo-plasma and thereby enables more effective charge transport than electrostatic induction. However, consistent with the physics of gas breakdown (Paschen’s law) (*31*), the reliance on gas ionization renders the mechanism intrinsically sensitive to environmental conditions. Specifically, lower ambient humidity and a gas with low ionization energy are conducive to further enhancing the electrical output of the bc-TENG (fig. S16). Despite this environmental dependency, a notable advantage of this mechanism lies in its remarkable material universality. Effective electrical outputs are achieved using a wide variety of materials, including conventional polymers (e.g., PTFE, PFA, and nylon), paper, fabric, metals, and semiconductors (fig. S17). This broad applicability is attributed to the underlying physical process, tribo-plasma emission, which is readily initiated at diverse sliding interfaces. Most importantly, when a semiconductor or metal is used as the slider, the bc-TENG exhibits a fundamentally distinct electrical behavior compared to dc-TENGs based on air breakdown or the tribovoltaic effect. In this configuration, the reciprocating motion generates a bidirectional current signal with alternating positive and negative peaks (fig. S18, A and B). This bidirectional characteristic stands in stark contrast to the unidirectional dc output of conventional dc-TENGs, further confirming that the proposed tribo-plasma emission mechanism represents a distinct principle for mechanical energy conversion.

### dc output of the rotary-mode bc-TENG

**Fig. 4.**
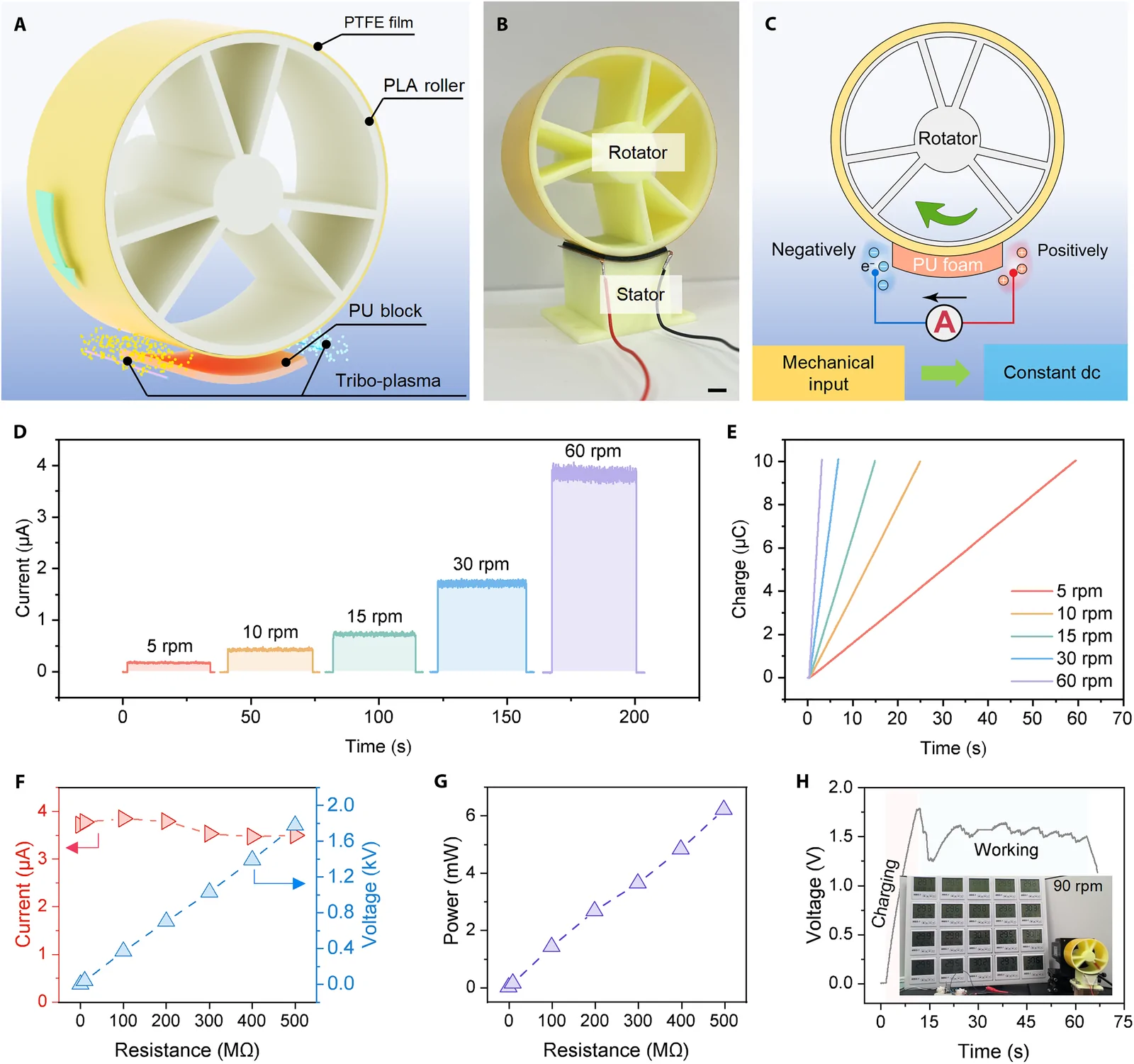
Design and power generation characteristics of the rotary-mode bc-TENG. (**A**) Three-dimensional schematic of the experimental setup for the rotary-mode bc-TENG, driven by a stepper motor. (**B**) Photographs of the fabricated rotator and stator components. Scale bar, 1 cm. (**C**) Circuit diagram illustrating the dc generation mechanism. (**D**) Output current waveforms at various rotation speeds (5 to 60 rpm), demonstrating the device’s constant-current characteristic. (**E**) Transferred charge over time at various rotation speeds, exhibiting a linear increase. (**F**) Dependence of the output current and voltage on the external load resistance. (**G**) Output power as a function of external load resistance, measured at a rotation speed of 60 rpm. (**H**) Charging curve of a 1-mF capacitor charged by the bc-TENG via a PMC while simultaneously powering 20 thermohygrometers (rotation speed: 90 rpm).

To achieve continuous and unidirectional energy conversion, we designed and fabricated a rotary-mode bc-TENG based on a roller structure (Fig. 4A). The device comprised a rotator coated with a PTFE film and a stator made of PU foam, flanked by two exposed copper electrodes that efficiently collect ions emitted from tribo-plasma (Fig. 4B). This low-cost architecture leverages the directional emission of tribo-plasma to produce a dc output (Fig. 4C and note S4). Probe measurements at two locations further support the origin of the dc generation, as sustained charge transfer is detected only within the tribo-plasma emission region (fig. S19). The inherent dc generation capability of the system was experimentally validated by its ability to directly power small electronics (e.g., a calculator), illuminate hundreds of light-emitting diodes (LEDs) (fig. S20 and movie S3), and charge various commercial capacitors (fig. S21). All of these demonstrations were performed without external rectification circuits. The electrical performance of the rotary-mode bc-TENG was systematically characterized. The *I*_SC_ shows a strong linear dependence on rotational speed, reaching 3.9 μA at 60 rpm (Fig. 4D and fig. S22). Correspondingly, the *Q* accumulates linearly over time at a constant speed, and its accumulation rate is directly proportional to the rotational speed (Fig. 4E). This behavior is attributed to the increased effective contact area per unit time at higher rotational speeds, which enhances the generation rate of the tribo-plasma. Moreover, owing to its high effective source impedance, the device can be approximated as a constant-current source, maintaining a stable output current over a wide range of load resistances (fig. S23). For instance, the root mean square current only decreases by 0.32 μA (8.3%) even as the external load resistance increases to 500 MΩ (Fig. 4F and fig. S24). This degree of current stability compares favorably with previously reported dc-TENGs (table S1). Consequently, this constant-current characteristic allows the output power (*P = I*^2^*R*) to increase monotonically with load resistance, resulting in a power of 6.2 mW at 500 MΩ (Fig. 4G). To evaluate the impedance matching characteristics of the bc-TENG, we measured its output power across a broad range of external load resistances. As illustrated in fig. S25, the device achieves a maximum output power of 17.2 mW at an optimal load resistance of 4 GΩ, translating to an equivalent average power density of 11.47 W/m^2^ per Hz. Benefiting from its favorable electrical performance and distinctive output characteristics, the rotary-mode bc-TENG shows promise for practical applications. When equipped with a power management circuit (fig. S26), a device with just 15 cm^2^ of effective contact area can continuously drive 20 commercial thermohygrometers at a rotation speed of 90 rpm (Fig. 4H and movie S4). Furthermore, the device facilitates contactless energy harvesting via the spatial propagation of tribo-plasma. Using a movable ion-collecting electrode positioned up to 10 mm away from the triboelectric interface, we demonstrated stable LED operation (movie S5), showing the feasibility of power delivery over macroscopic distances through ambient ion collection. Collectively, these attributes underscore the device’s potential as a compact and efficient power source for low-power electronics, thereby enabling its future integration into distributed energy systems.

### Direct applications of in situ tribo-plasma

**Fig. 5.**
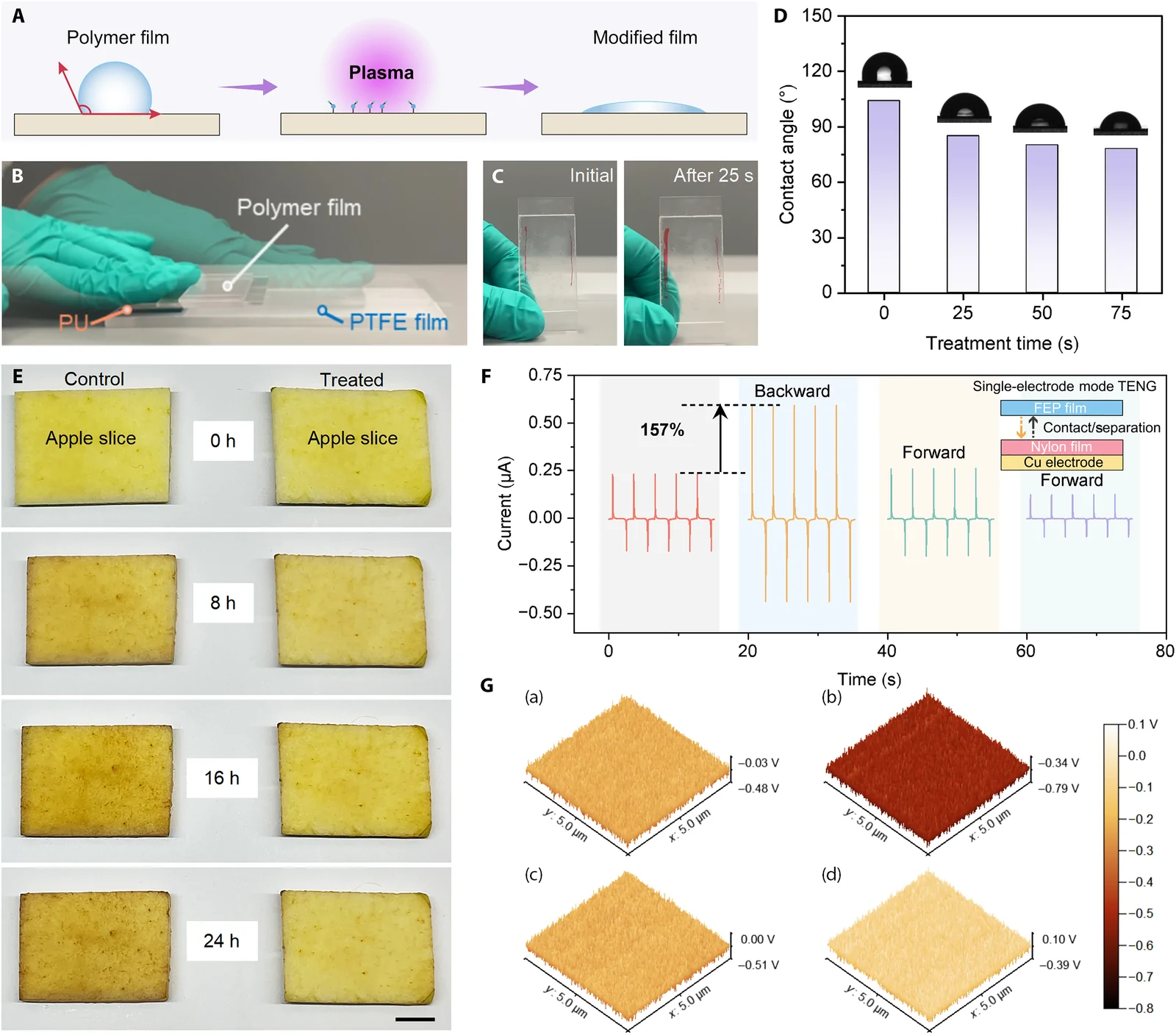
Demonstrations of in situ tribo-plasma applications. (**A**) Schematic illustrating the mechanism of plasma-induced surface modification for enhanced wettability. (**B**) Photograph of the experimental setup used for the in situ tribo-plasma treatment of an FEP film. (**C**) Photographs comparing ink adhesion before and after 25 s of treatment. (**D**) Water contact angle measured versus treatment time. (**E**) Photographs of apple slices at the initial state (0 hours) and after 8, 16, and 24 hours of exposure for the control and tribo-plasma–treated groups. (**F**) Output current from a single-electrode TENG based on an FEP active layer that underwent sequential negative and positive ion implantation. (**G**) Corresponding surface potential mapping images.

The applications of in situ tribo-plasma generation extend far beyond energy harvesting, offering a versatile and power-free technological platform for diverse processes, including polymer surface modification (*32*, *33*), food preservation (*34*, *35*), and ion implantation (*36*). As a demonstration of this capability, we first used the tribo-plasma for polymer surface modification. Plasma-based surface engineering is widely used to enhance material hydrophilicity by introducing oxygen-containing functional groups (Fig. 5A). Here, we demonstrate that the plasma spontaneously generated in situ during sliding contact effectively enables surface modification. To validate this approach, a commercial fluorinated ethylene propylene (FEP) film was mounted at the edge of the PU slider (Fig. 5B), with a 2-mm air gap from the opposing PTFE surface. After only 25 s of sliding, the FEP film exhibited an obvious transition in wettability. As depicted in Fig. 5C, the initially hydrophobic surface became readily wettable, evidenced by its ability to be marked with ink (movie S6). This surface modification was further quantified by static water contact-angle measurements, which revealed a substantial decrease from an initial ∼104° to ∼78° after a treatment period of 75 s (Fig. 5D). These results establish tribo-plasma as a rapid and effective method for hydrophilic surface modification without requiring vacuum systems or chemical reagents.

Second, plasma techniques are effective for food preservation by suppressing enzymatic browning. To validate this potential, a proof-of-concept experiment was performed using two freshly cut apple slices. As illustrated in Fig. 5E, the right sample treated with tribo-plasma for ∼10 min exhibits markedly inhibited browning, retaining a fresher appearance. By contrast, the untreated control sample (left) undergoes discoloration under identical conditions. Consistent with this visual observation, after 24 hours of exposure, the quantitative analysis indicates that the change in the CIELAB parameter b* is substantially smaller for the treated sample (Δb* = 2.75) than for the control (Δb* = 6.49), where a smaller Δb* reflects a weaker shift toward yellowing/browning (fig. S27A). In addition, the L* value (representing lightness/brightness) is better maintained after tribo-plasma treatment (fig. S27B). This preservation effect is attributed to the inactivation of key enzymes, such as polyphenol oxidase (*37*), by reactive oxygen and nitrogen species within the plasma. This process extends the shelf life and sensory appeal of fresh fruit without chemical additives. Last, we leverage the polarity-selective emission of charged particles from the tribo-plasma to develop a power-free ion implantation strategy. As dictated by the underlying physical mechanism, the forward and backward motions of the slider correspond to the emission of predominantly positive and negative ions, respectively. To validate this principle, we used a single-electrode mode TENG as an in situ probe to monitor the surface charge of a target FEP film (the inset in Fig. 5F). The *I*_SC_ and *Q* results confirm a highly controllable ion implantation process (Fig. 5F and fig. S28), where the polarity of the injected ions is determined solely by the sliding direction. Specifically, forward (rightward) sliding followed by a noncontact return stroke injects positive ions, whereas initial backward (leftward) sliding injects negative ions. This precise control is demonstrated through a three-stage experiment. First, 50 backward sliding strokes induce negative ion implantation (fig. S29A), causing the TENG’s output current to increase by 157% from a baseline of 0.23 to 0.59 μA (Fig. 5F). Subsequently, 50 forward strokes emit positive ions that progressively neutralize the preimplanted negative charges (fig. S29B), restoring the current to its initial level. Last, an additional 20 forward strokes lead to an accumulation of positive charges, which suppresses the TENG output below its initial value. To substantiate the surface-charge modulation mechanism, Kelvin probe force microscopy (KPFM) measurements were performed (Fig. 5G). The pronounced shift in surface potential before and after the treatment correlates well with the observed trend in current variation. Specifically, negative-ion implantation induces a substantial negative shift in surface potential relative to the pristine state [Fig. 5G (a and b)]. This controlled and bidirectional charge modulation validates the polarity-selective nature of tribo-plasma emission and offers a route toward in situ ion implantation that is simple and entirely independent of external high-voltage power sources.

## DISCUSSION

In summary, this work presents and validates an approach for triboelectric energy conversion based on tribo-plasma emission. The core of this approach is to leverage the intrinsic triboelectric field between sliding dielectrics. This field enables a seamless coupling between in situ plasma generation and its directional emission during mechanical motion. In the present system, tribo-plasma originates from spontaneous ESD between sliding dielectric materials. Furthermore, the inherently asymmetric electric-field distribution within the sliding system provides the driving force for efficient plasma separation and collection. Based on this mechanism, the developed bc-TENG is capable of delivering either a quasi-square-wave ac output or a constant dc output depending on the motion profile. To optimize device performance and guide material selection, 20 distinct material pairs were systematically characterized and ranked to establish a triboelectric series. Beyond energy harvesting, we further demonstrate that the in situ plasma can be directly used for applications such as polymer surface modification, food preservation, and directional ion implantation without any external power source. Collectively, this study introduces a potential route for efficient mechanical energy conversion and establishes the theoretical and experimental foundation for passive plasma technologies, thereby substantially broadening the scientific and technological scope of triboelectrification.

## MATERIALS AND METHODS

### Fabrication of the sliding-mode bc-TENG

Slider and electrodes: The slider fabrication commenced with the laser cutting of an acrylic sheet (50 mm by 30 mm by 3 mm, length × width × thickness) to serve as the substrate and a PU sheet (50 mm by 30 mm by 2 mm) for the triboelectric layer. The PU sheet was then bonded to the acrylic substrate. For the electrode assembly, four polyethylene terephthalate (PET) sheets (50 mm by 30 mm by 0.5 mm) were prepared, and a shallow groove (50 mm by 5 mm) was engraved into the surface of each. Copper foil tape was subsequently laminated onto the grooved area of all four PET sheets. To create the insulated electrodes, two of these copper-laminated sheets were coated with an electronic-grade epoxy potting compound, fully encapsulating the copper foil. The remaining two served as the exposed electrodes. Last, the two exposed and two insulated electrodes were mounted onto three-dimensional (3D) printed brackets, which allowed for precise height adjustment and alignment.

Stator: The stator was fabricated by attaching various dielectric films (120 mm by 90 mm), including PTFE, perfluoroalkoxy alkane, nylon, and paper, onto laser-cut acrylic substrates (120 mm by 90 mm by 5 mm).

### Fabrication of the rotary-mode bc-TENG

Stator: The stator was constructed from a PET substrate (50 mm by 34 mm by 0.5 mm). Initially, two parallel shallow grooves were engraved on its surface. A PU sheet (50 mm by 30 mm by 2 mm), acting as the primary triboelectric layer, was then bonded to the central region of the PET substrate. Subsequently, copper foil tape was laminated onto the remaining exposed areas on both sides of the PU layer, forming two distinct electrodes separated by the grooves. Last, copper wires were electrically connected to each copper foil electrode for signal output.

Rotator: The rotator was fabricated by 3D printing a cylindrical core (Φ 84 mm by 50 mm). A PTFE film (263.8 mm by 50 mm, 0.05-mm thick) was then securely wrapped around and bonded to the outer surface of the printed core.

### Tribo-plasma treatment of apple slices

Two fresh apple slices (3 cm by 4 cm, ∼5-mm thick) was secured onto a custom-fabricated bracket rigidly attached to the PU slider. This configuration enabled the sample to undergo synchronous reciprocating motion with the slider, ensuring continuous exposure to the in situ generated tribo-plasma. The treatment was conducted for 10 min with a stroke length of 100 mm, while a constant vertical gap of 5 mm was maintained between the exposed apple slice surface and the underlying PTFE film.

### Measurement and characterization

Dark-field optical images were acquired using a smartphone camera (Huawei P40 Pro). The concentration of ambient air ions was monitored with an air ion counter (WST-09DF). For electrical characterization, the short-circuit current and transferred charge were measured by a programmable electrometer (Keithley 6514), while the output voltage was recorded using a high-impedance electrostatic voltmeter (Trek 370). A linear motor (LinMot S01-72/500) and a stepper motor (86HB250-118B) were used to drive controlled linear and rotational motion, respectively. A customized data acquisition system, implemented with LabVIEW and a data acquisition card (NI-9235), was used for synchronized data logging. All finite-element simulations were performed using COMSOL Multiphysics software. The humidity was maintained at ∼35% RH during the measurements. KPFM measurements were conducted using a Park NX20 atomic force microscope equipped with an NSC36/Cr-Au-C conductive probe. The measurements were performed at a nonresonant frequency of 17 kHz, with a 2-V ac drive and a phase of −51.56°. Images were acquired over an area of 5 μm by 5 μm at a scan rate of 0.5 Hz.

## Supplementary Materials

### The PDF file includes:

Figs. S1 to S29 Notes S1 to S4 Tables S1 and S2 Legends for movies S1 to S6 References
