# Supplementary material for: Harnessing in-situ tribo-plasma emission for energy harvesting
Source: Sci Adv. 2026 Sep 9;12(37):eaei0397. doi: 10.1126/sciadv.aei0397 (PMC13557092; doi:10.1126/sciadv.aei0397)
Supplement: Supplementary file 1 [file sciadv.aei0397_sm.pdf]

Science Advances
AAAS

Supplementary Materials for

Harnessing in-situ tribo-plasma emission for energy harvesting

Qixuan Zeng *et al.*

Corresponding author: Xue Wang, xuewang@cqu.edu.cn

Sci. Adv. **12**, eaei0397 (2026)
DOI: 10.1126/sciadv.aei0397

The PDF file includes:

Figs. S1 to S29
Notes S1 to S4
Tables S1 and S2
Legends for movies S1 to S6
References

Other Supplementary Material for this manuscript includes the following:

Movies S1 to S6

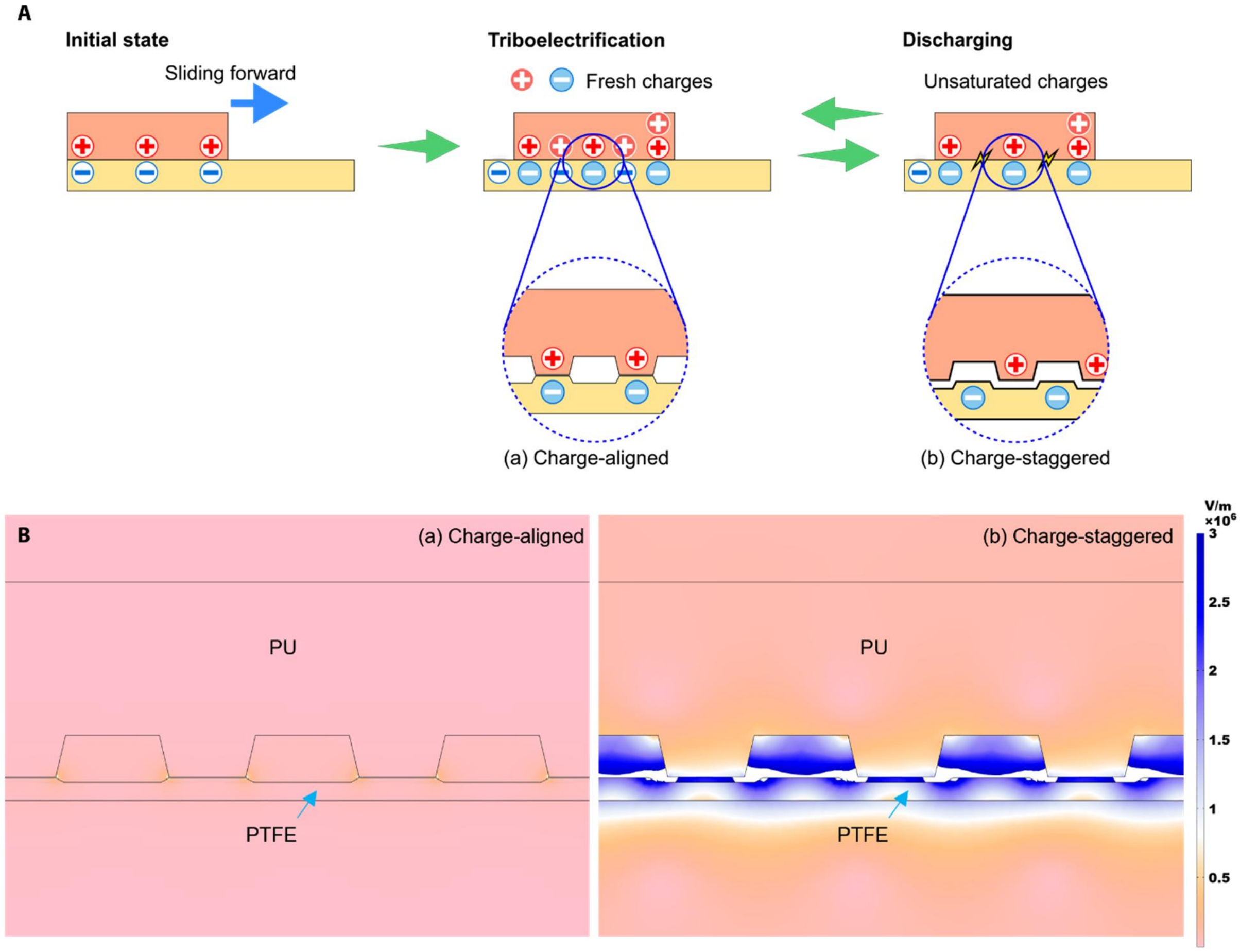

Fig. S1 | Dynamic processes of triboelectrification and discharge at the PU-PTFE interface. (A) Schematic illustration of the dynamic processes of triboelectrification and discharge at the PU-PTFE interface. (B) Finite element simulation results of the electrostatic field strength at the gap under (a) charge-aligned and (b) charge-staggered conditions. The blank areas indicate that the electrostatic field strength exceeds the dielectric strength of air (3 kV/mm).

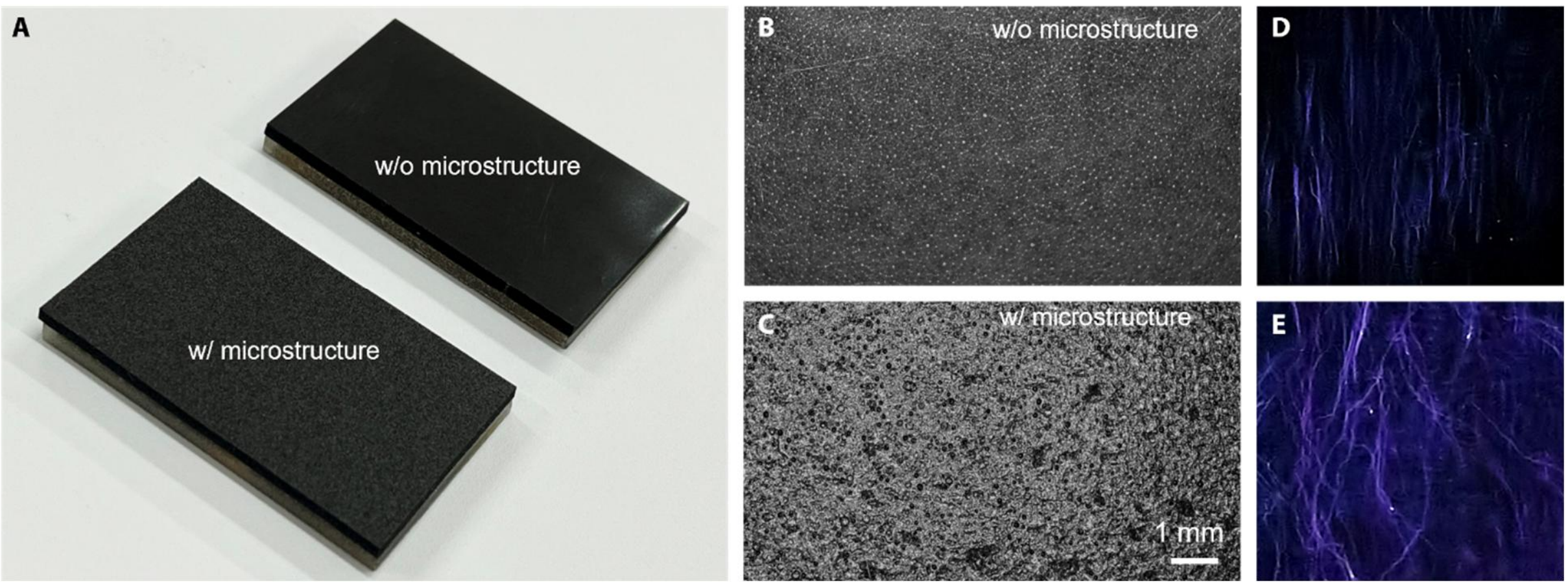

Fig. S2 | Comparative analysis of PU sliders with distinct surface morphologies. (A) Optical photographs of the two PU blocks. (B, C) Enlarged views of the PU surfaces without and with microstructures, respectively. (D, E) Photographs of interfacial discharge during sliding for the PU surfaces without and with microstructures, respectively.

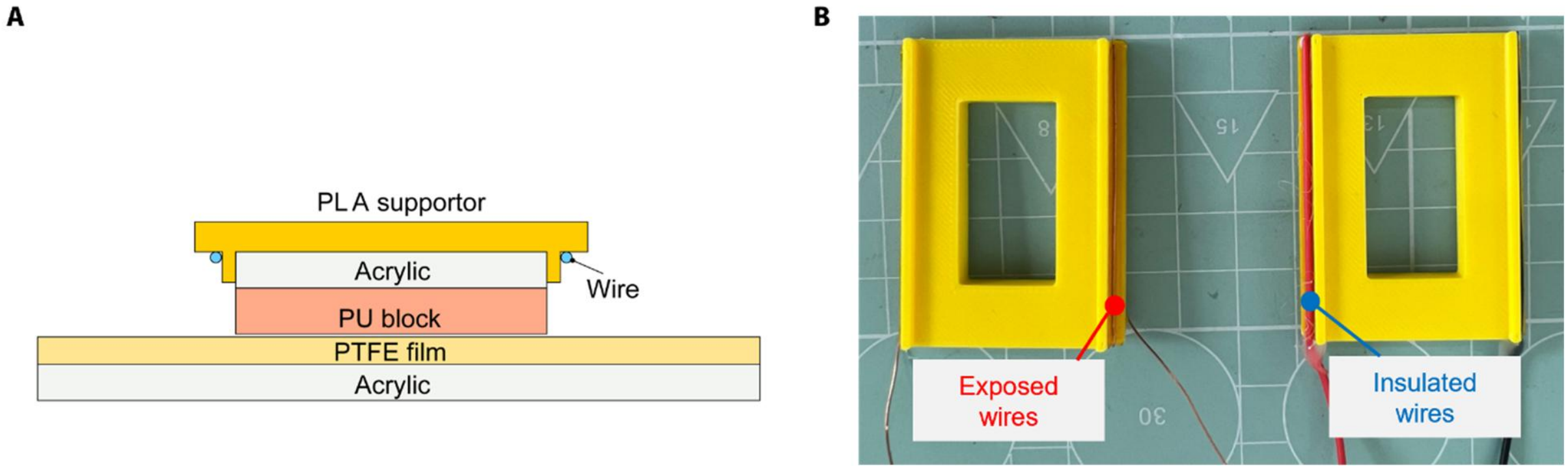

Fig. S3 | Architecture and components of the BC-TENG. (A) Schematic of the BC-TENG, illustrating the configuration of exposed and insulated wires mounted on a PLA support frame. (B) Photograph of the configurations of the exposed and insulated wires.

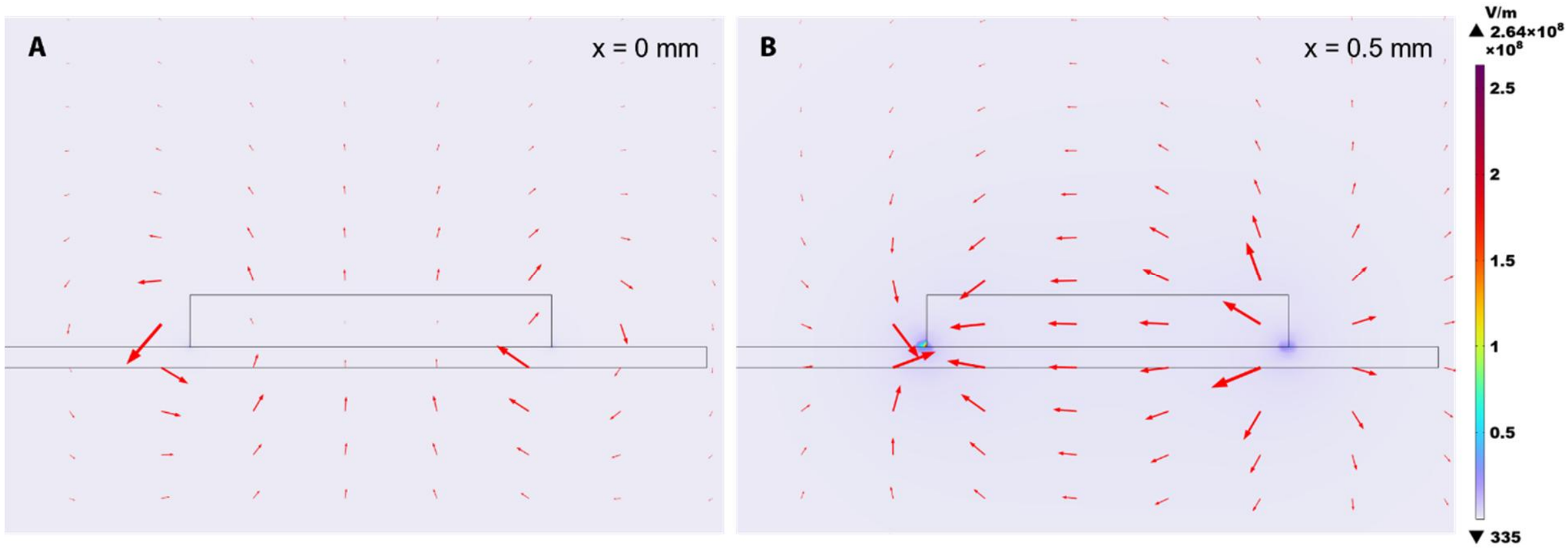

Fig. S4 | The distribution of the electric field pattern. (A) Initial position (x = 0 mm). (B) 0.5 mm forward displacement (x = 0.5 mm).

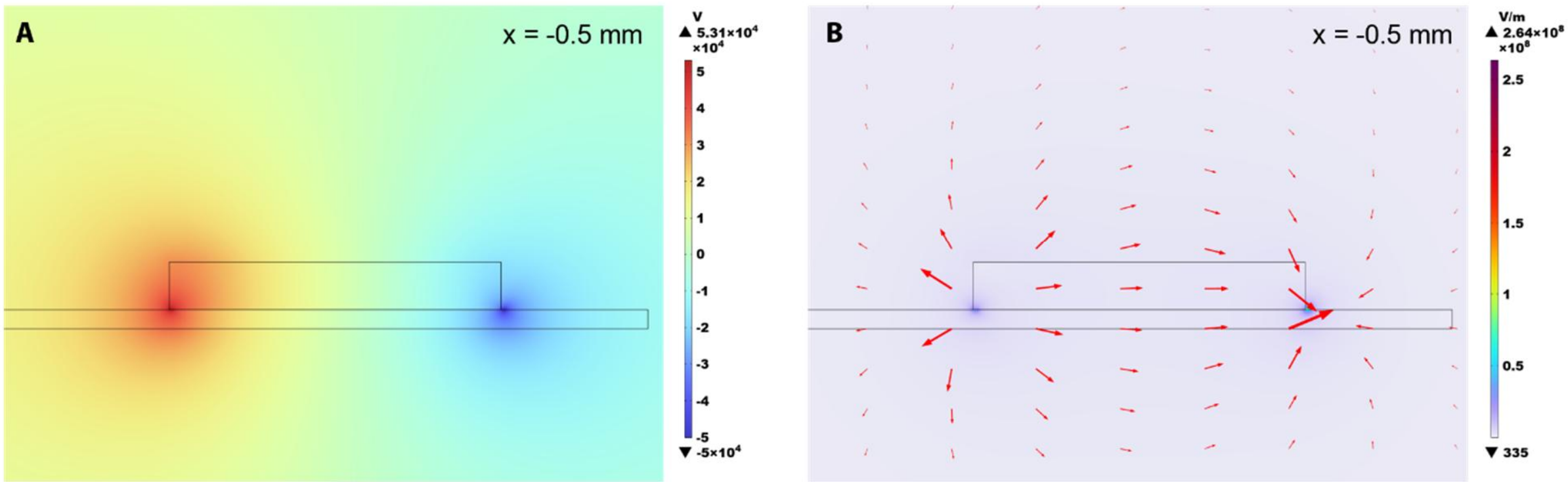

Fig. S5 | Simulated distributions of electric potential and electric field. (A) Electric potential and (B) Electric field distributions for the slider moved backward (x = -0.5 mm).

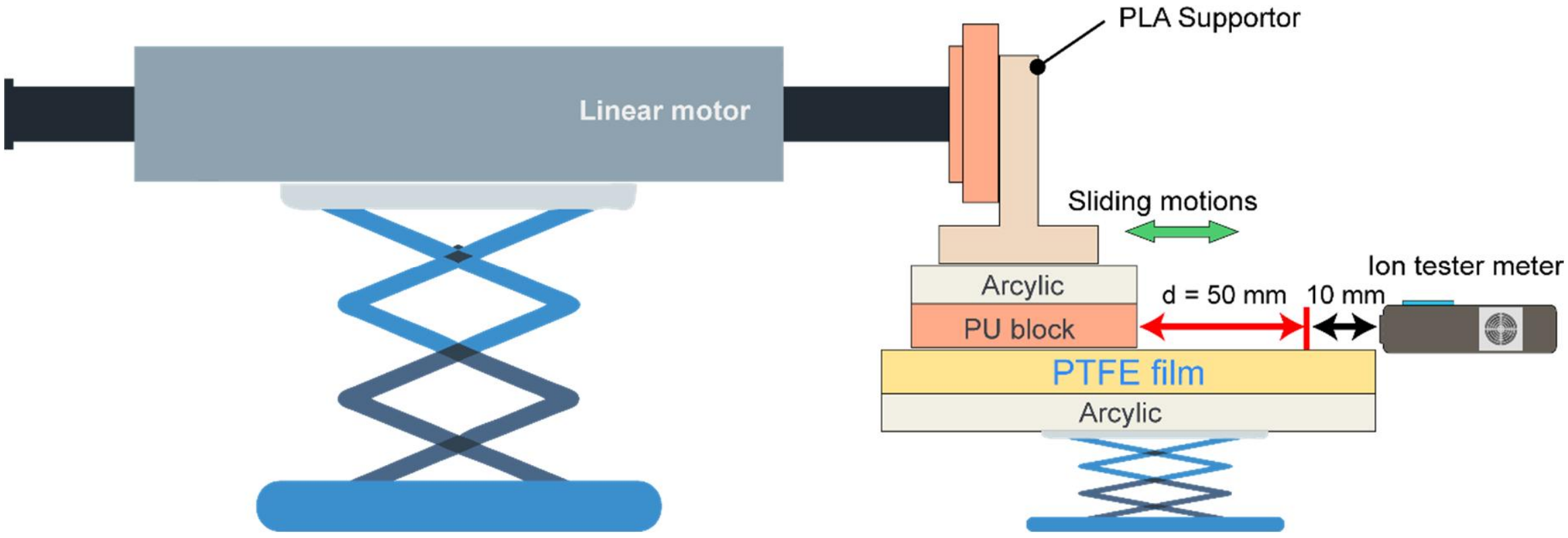

Fig. S6 | Ion concentration measurement platform. The ion counter's inlet is positioned horizontally above the PTFE surface and oriented towards the slider. A minimum separation distance of 10 mm is established when the slider reaches the rightmost endpoint of its 50 mm reciprocating stroke.

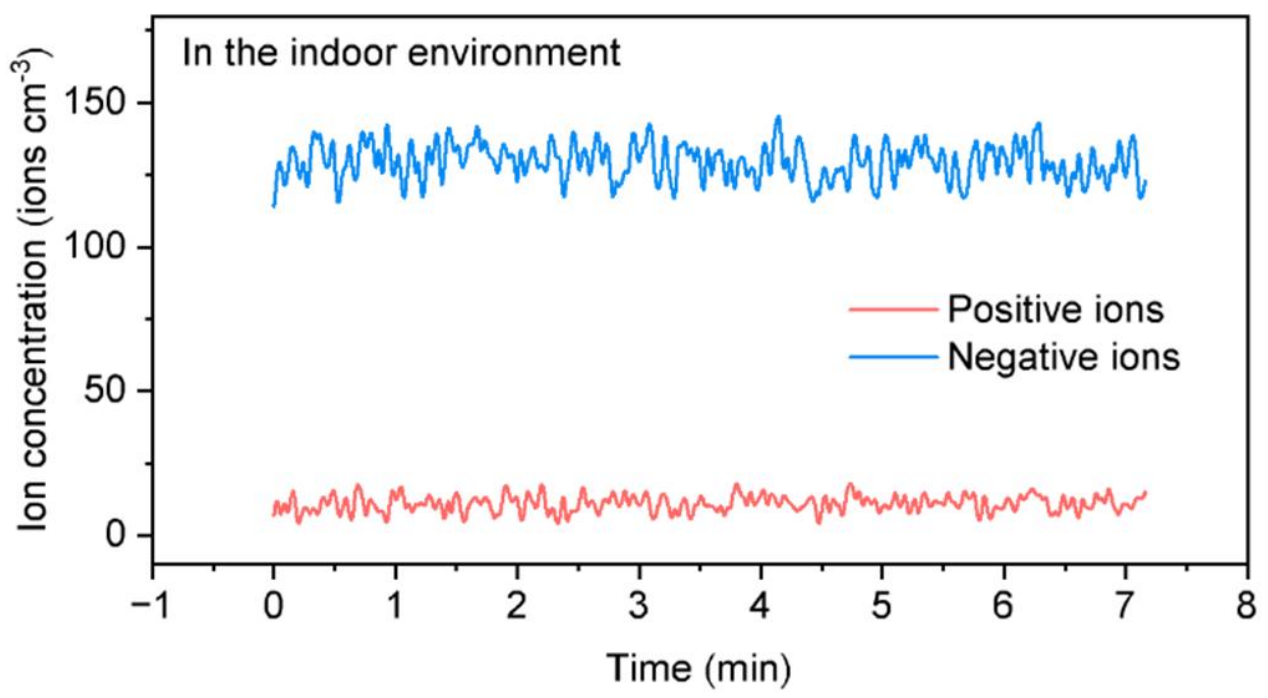

Fig. S7 | Ion concentration measurement of positive and negative ions under static condition. The 7-minute measurement profile confirms that the indoor ambient air ion concentration was maintained at a low and stable level.

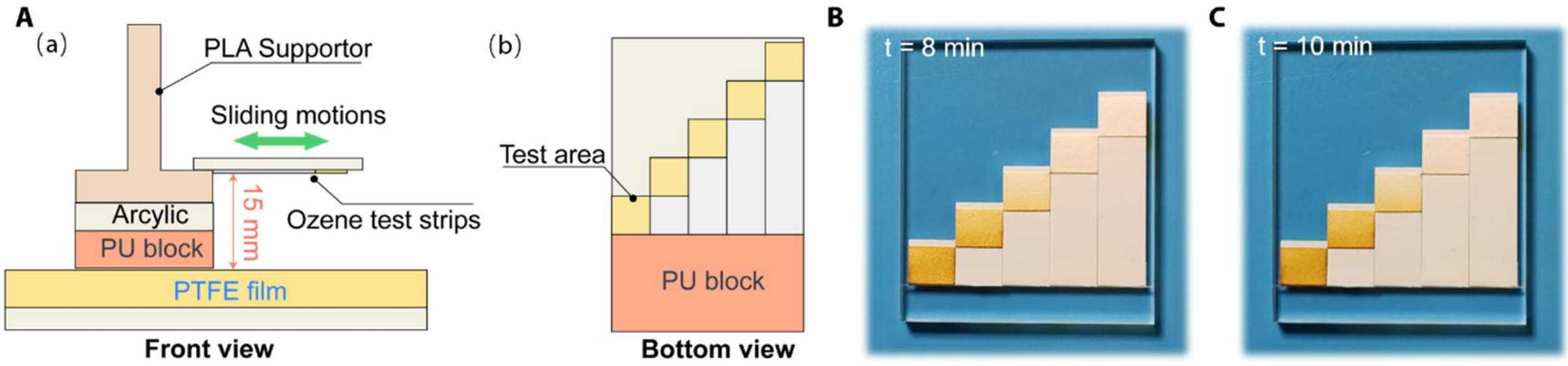

Fig. S8 | Mapping the spatial distribution of tribo-plasma via ozone detection. (A) Schematic of the ozone monitoring setup; (a) front view and (b) bottom view. (B, C) Photographs of the color change of the ozone test strips after 8 and 10 min, respectively.

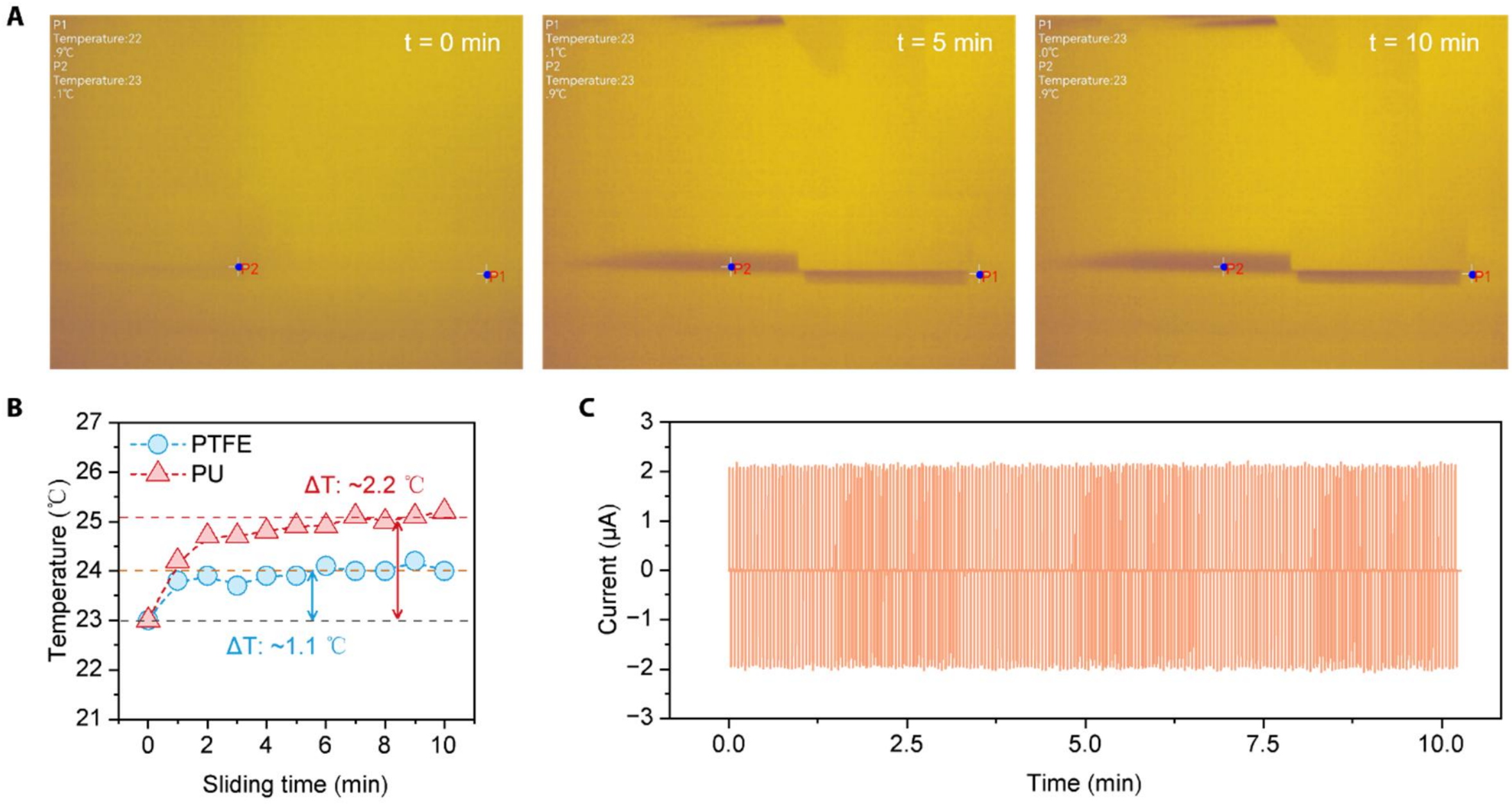

Fig. S9 | Temperature variation and output stability. (A) Infrared thermal images of the triboelectric materials after different sliding durations (0, 5, and 10 min). (B) Temperature evolution of the PU block and PTFE film as a function of sliding time. (C) Current output waveform during continuous sliding for 10 min.

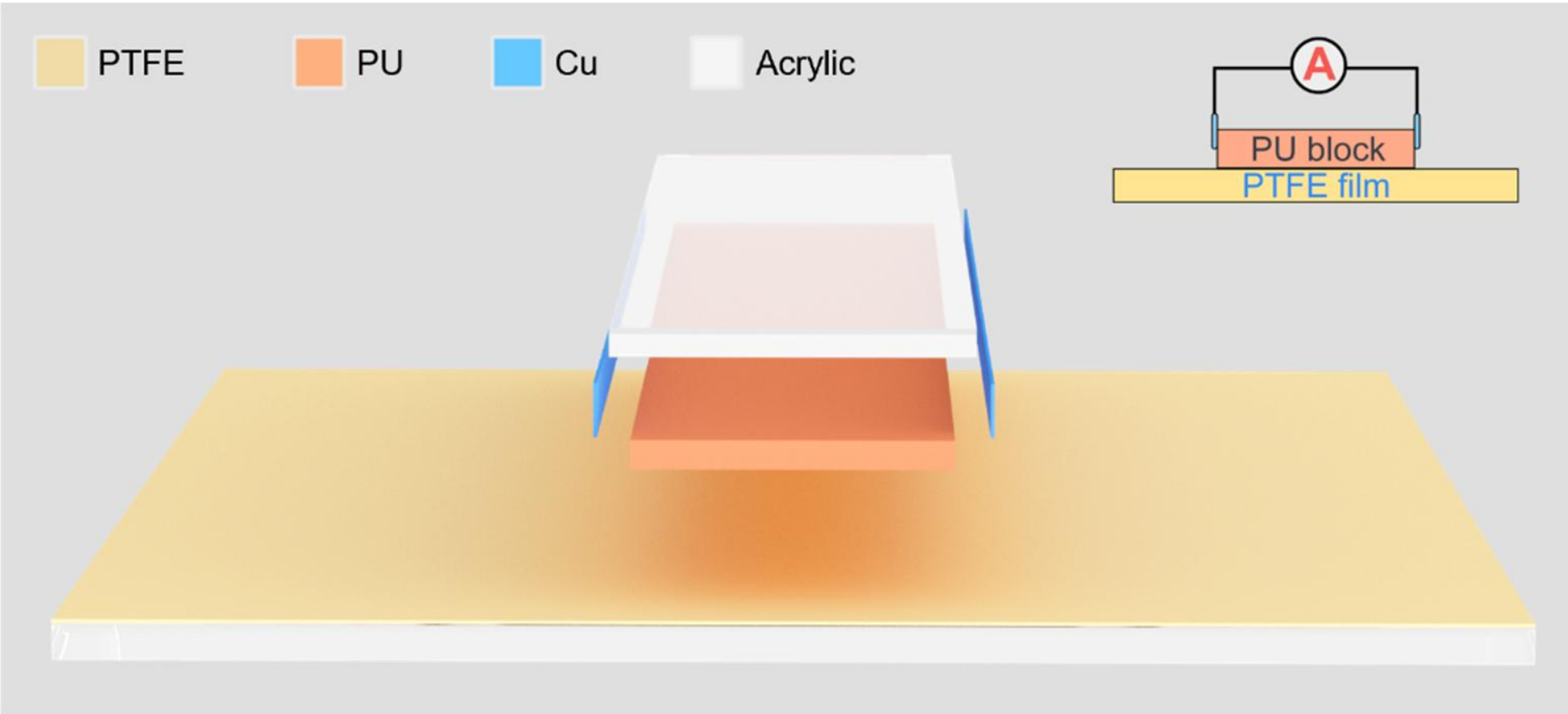

Fig. S10 | Device architecture of the sliding-mode BC-TENG. The main panel provides a 3D schematic illustrating the detailed structure of the device. The inset shows the electrical measurement configuration used for performance characterization.

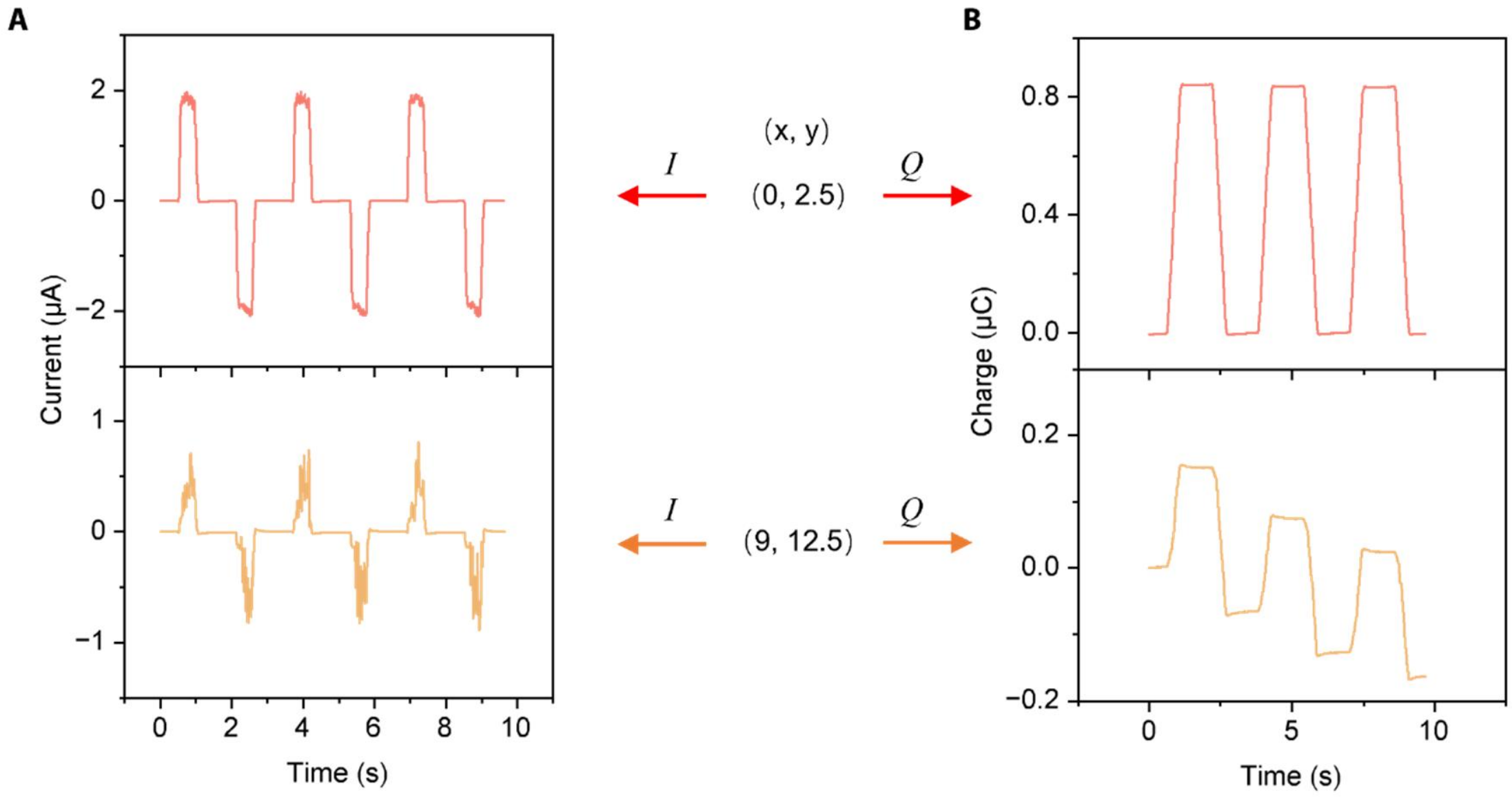

Fig. S11 | Comparison of the electrical output at the two extremal positions. Temporal profile of the (A) output current and (B) the transferred charge.

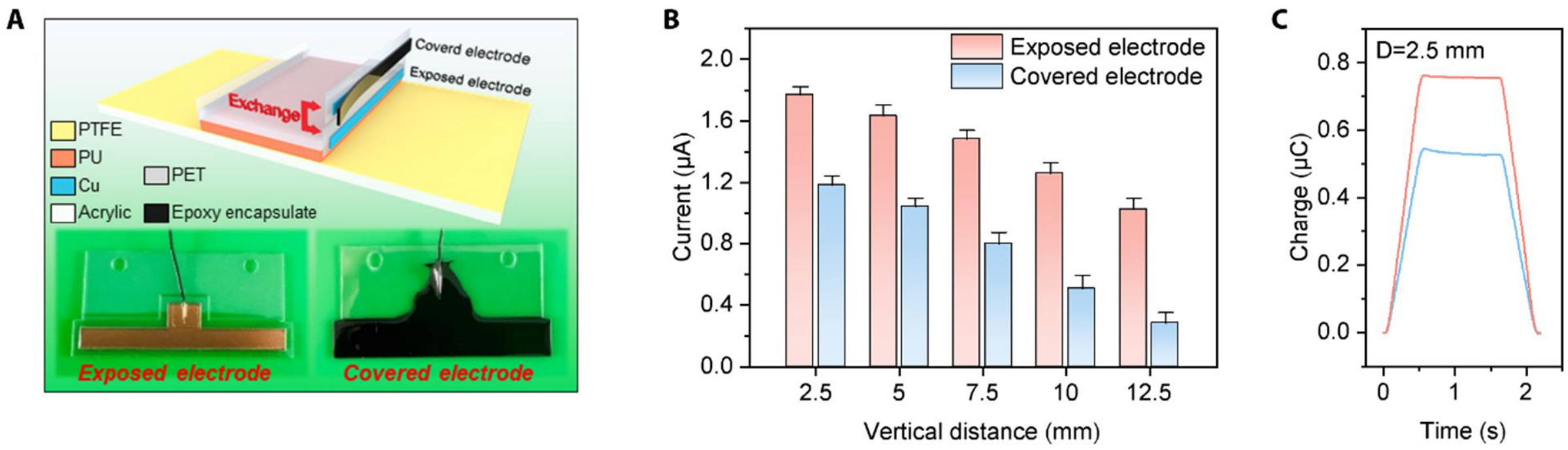

Fig. S12 | Performance comparison of exposed versus covered electrodes. (A) 3D schematic illustrations and corresponding physical photographs of the two electrode configurations. The covered electrodes were fabricated by encapsulating the exposed ones with an insulating resin. (B) Comparison of the output currents as a function of vertical separation distance. (C) Comparison of the transferred charge profiles at a fixed vertical distance of D = 2.5 mm.

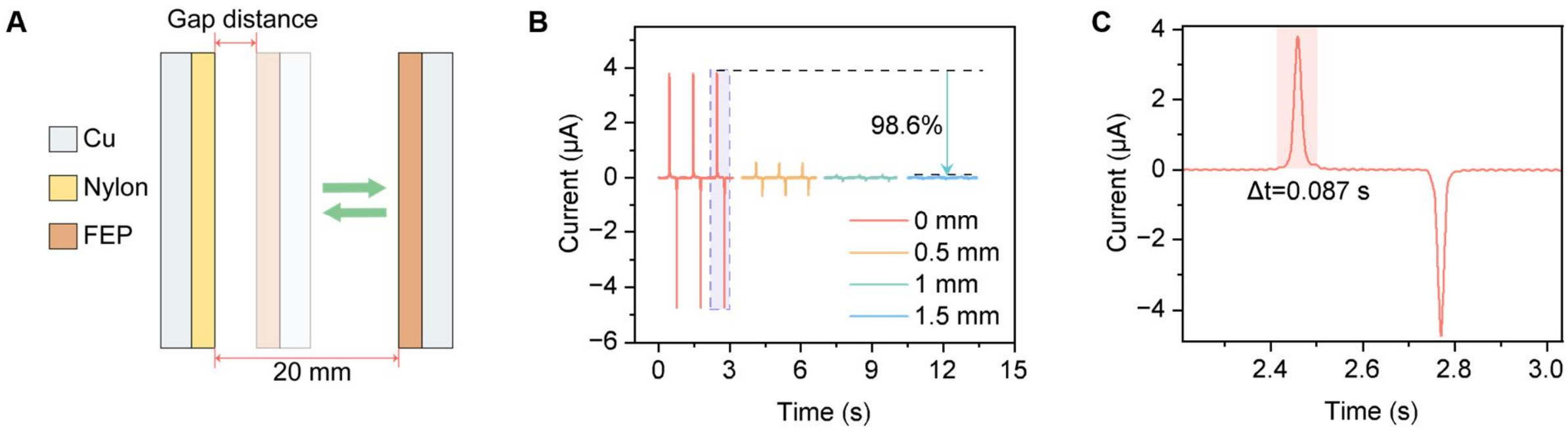

Fig. S13 | Electrical output characteristics of the CS-TENG. (A) Schematic model of the CS-TENG with a maximum separation displacement of 20 mm. (B) Output current waveforms at different gap distances. (C) Enlarged view of the current waveforms within the purple-shaded region in (B).

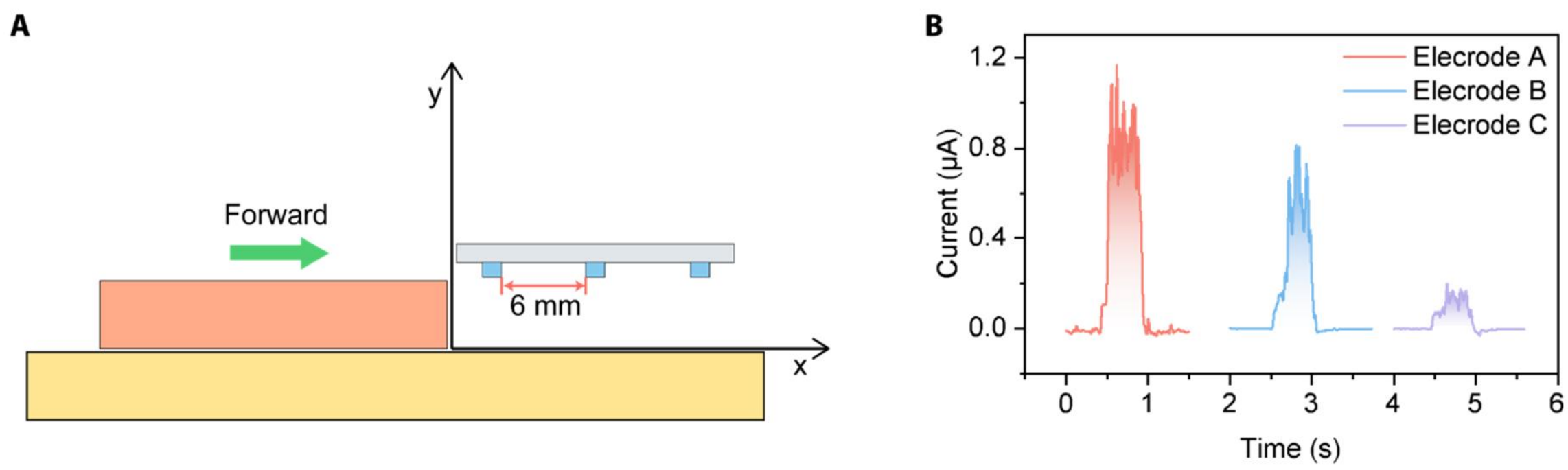

Fig. S14 | Performance of the BC-TENG featuring a multi-electrode array. (A) Schematic of the BC-TENG with a multi-electrode array (Each electrode has a width of 2 mm, with an inter-electrode spacing of 6 mm, and is positioned 10 mm above the bottom PTFE film). (B) Current signals generated by electrodes A, B, and C during the forward movement of the PU slider.

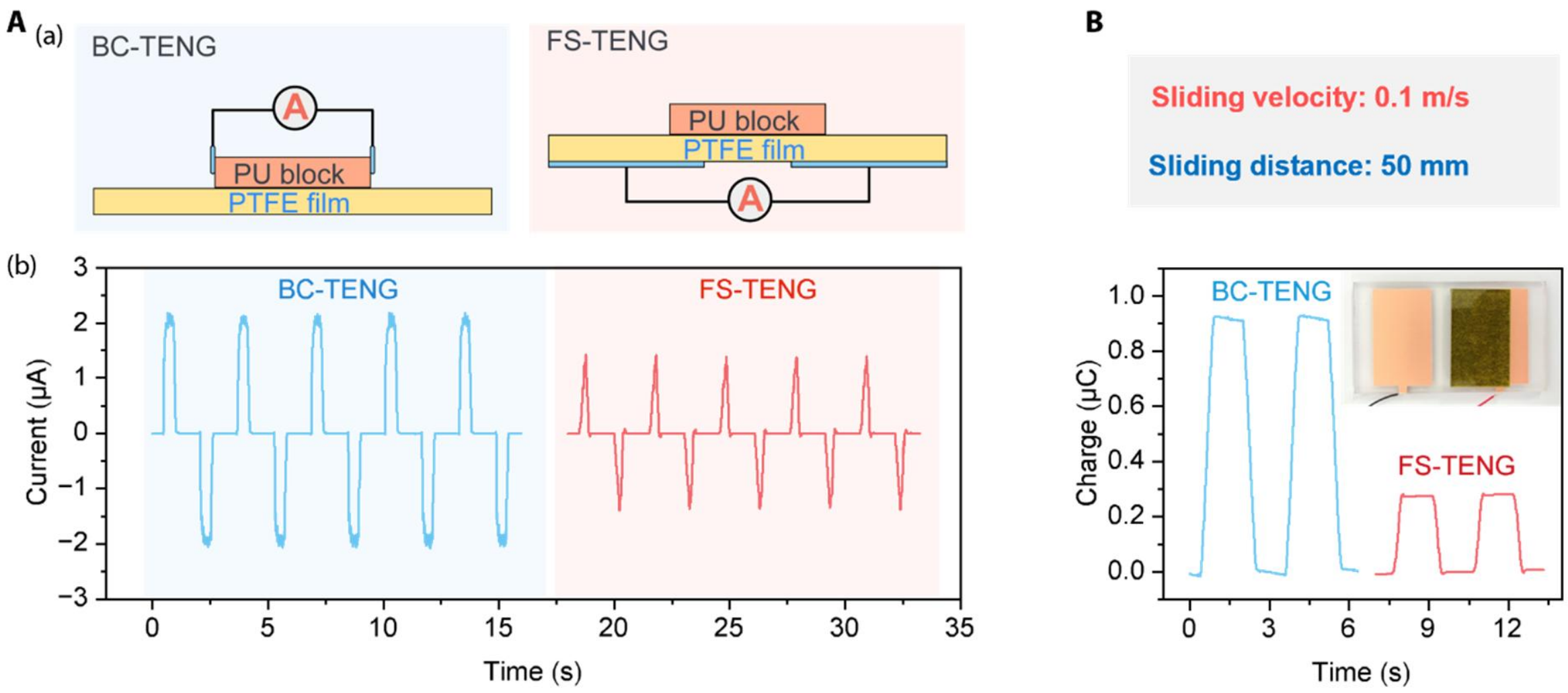

Fig. S15 | Comparative analysis of the BC-TENG and a conventional FS-TENG. (A) Schematic illustrations of device architectures and corresponding output currents. (B) Comparison of transferred charge.

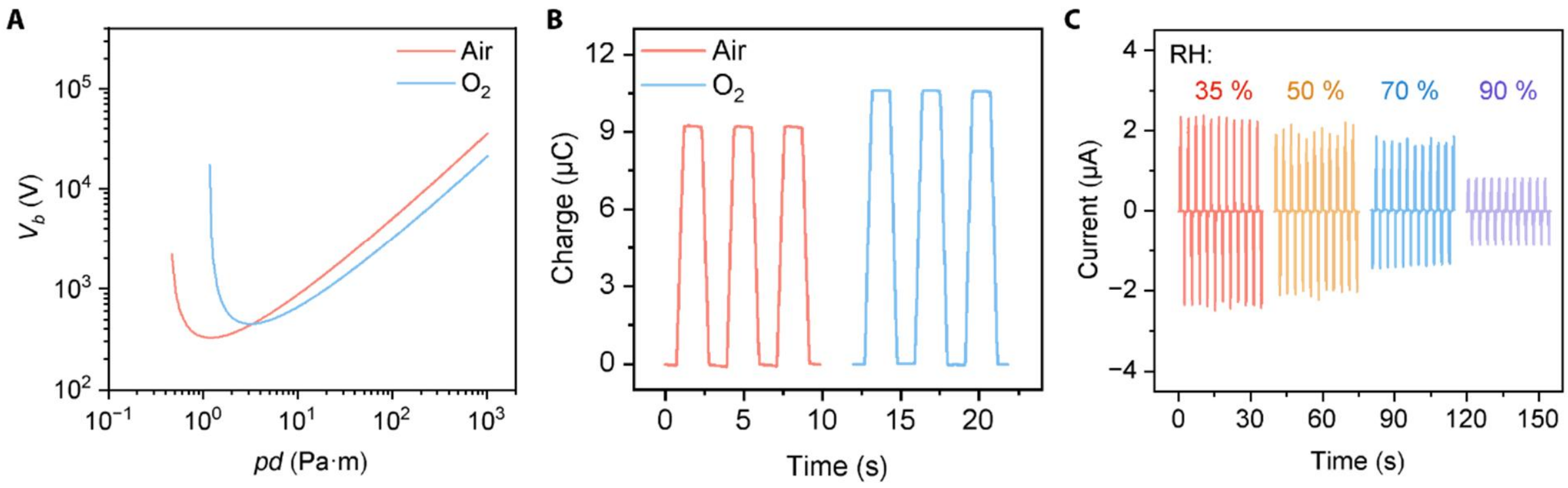

Fig. S16 | Output performance of the BC-TENG under different gas atmospheres and humidity levels. (A) Paschen curves in air and O_2. (B) Transferred charge curves in air and O_2. (C) Current output under different relative humidity conditions.

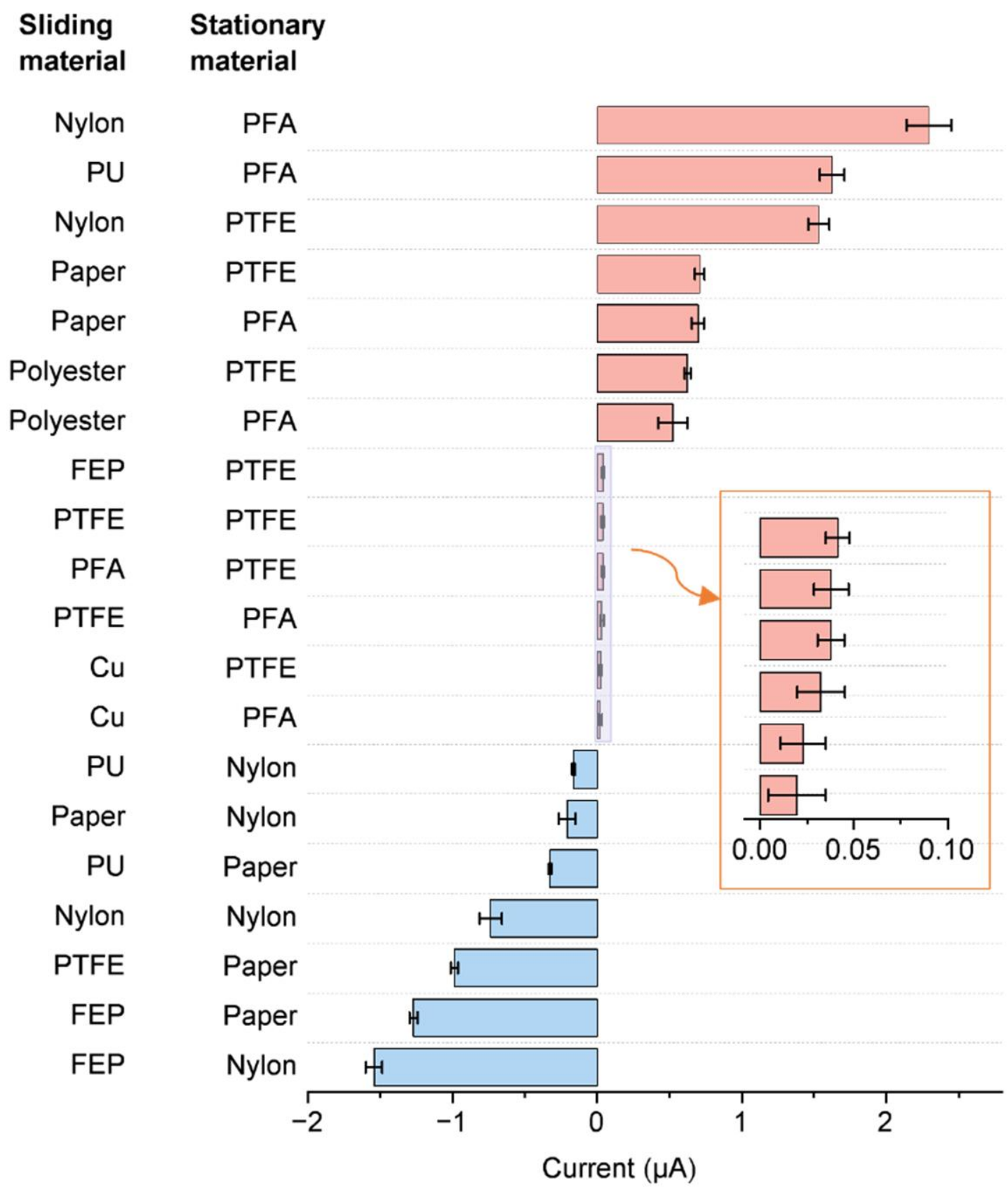

Fig. S17 | Output current amplitude for various triboelectric material pairs. This figure compares the current amplitude generated from different sliding materials paired with stationary materials. The current's polarity (positive or negative) indicates its direction during rightward sliding and is determined by the relative electronegativity of the two materials in contact.

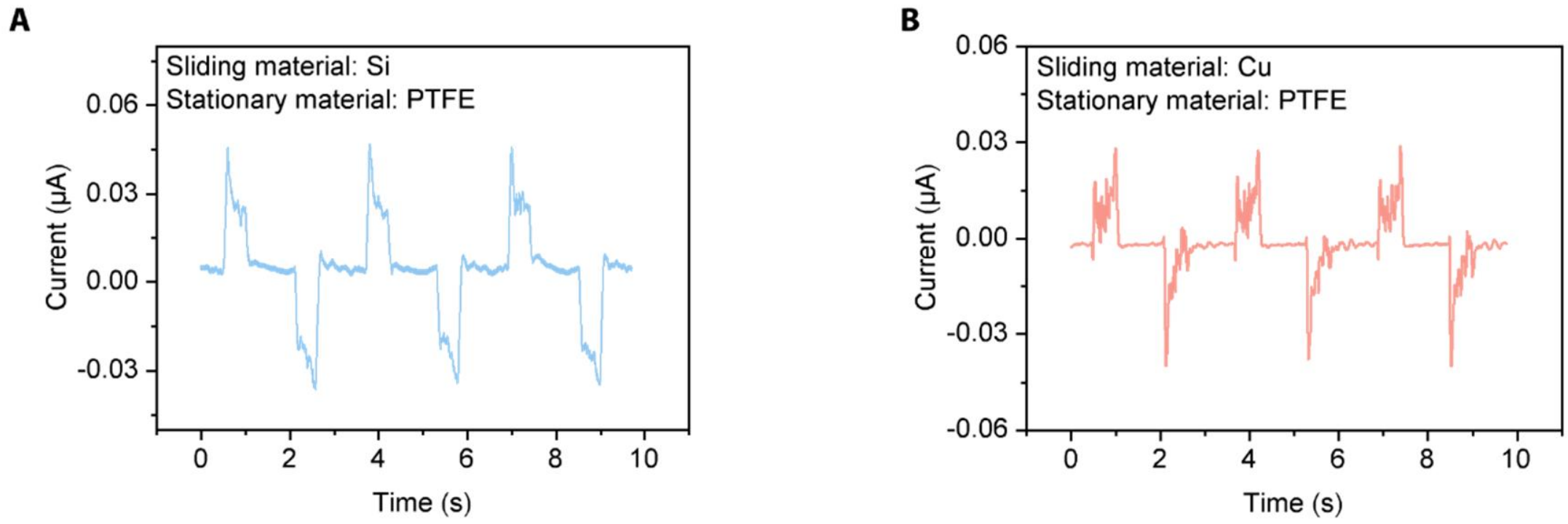

Fig. S18 | Output currents from semiconductor and metallic sliders. (A) Measured current profile for a Si slider. (B) Measured current profile for a Cu slider.

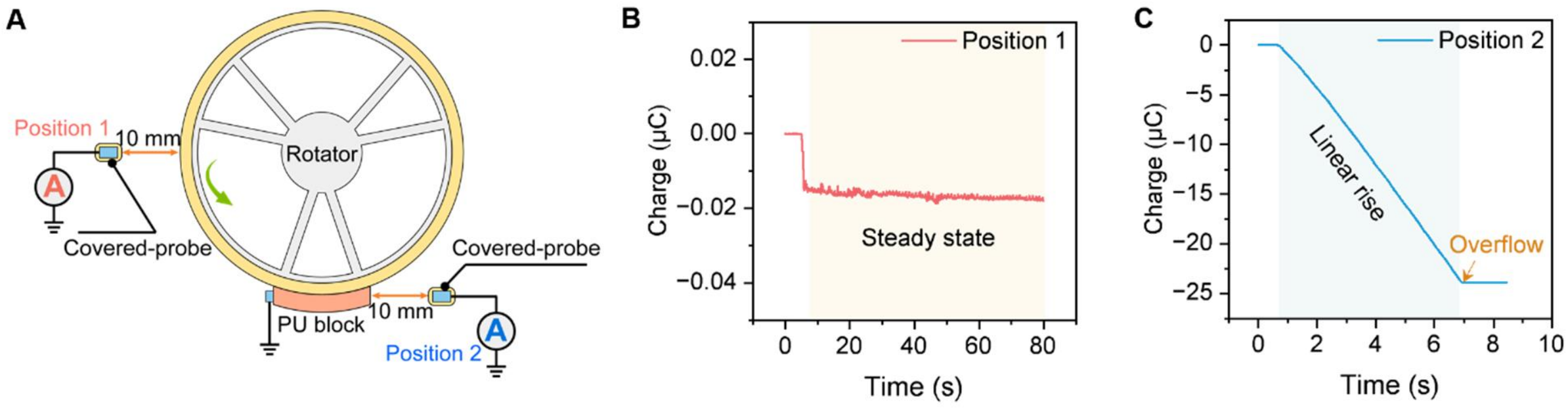

Fig. S19 | Measurement of transferred charge at different positions. (A) Schematic illustration of the measurement setup. (B, C) Transferred-charge curves measured at Positions 1 and 2.

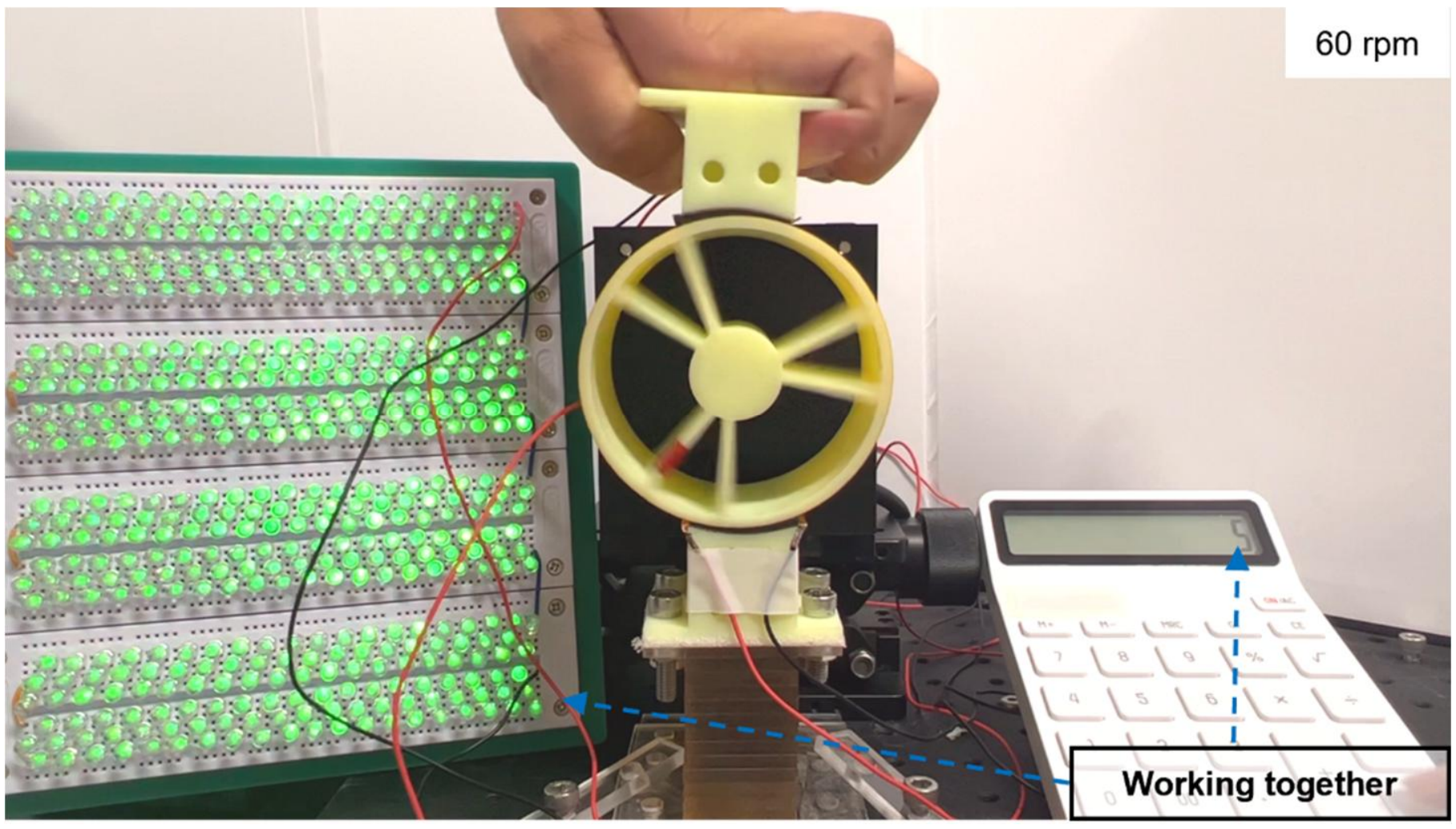

Fig. S20 | Demonstration of the BC-TENG simultaneously powering a calculator and illuminating LEDs.

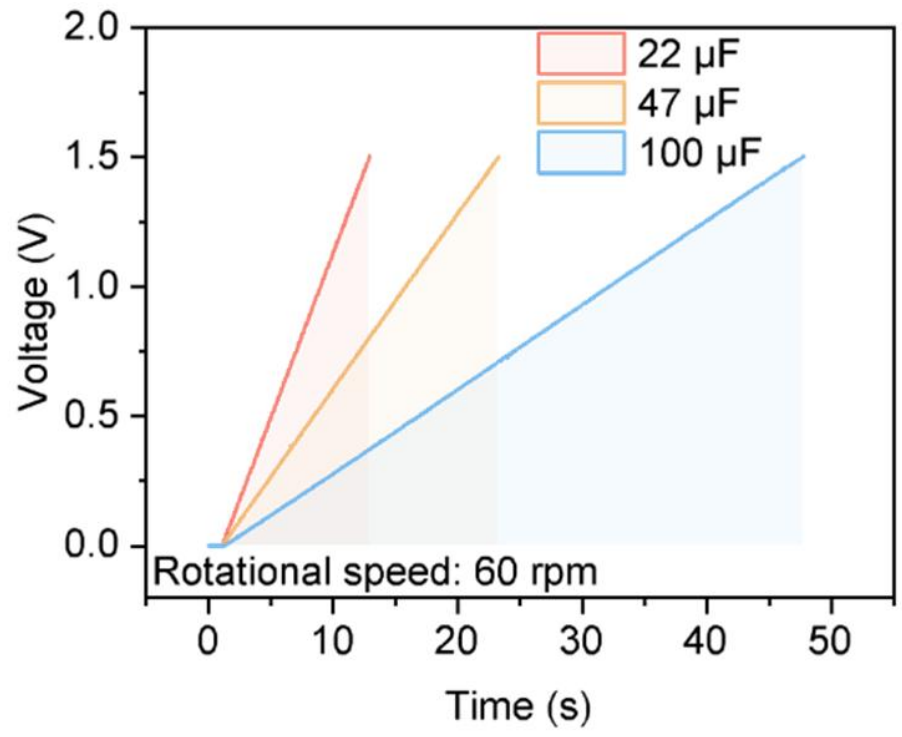

Fig. S21 | Charging profiles of various capacitors powered by the BC-TENG. The BC-TENG is capable of directly charging capacitors, eliminating the need for a rectifier.

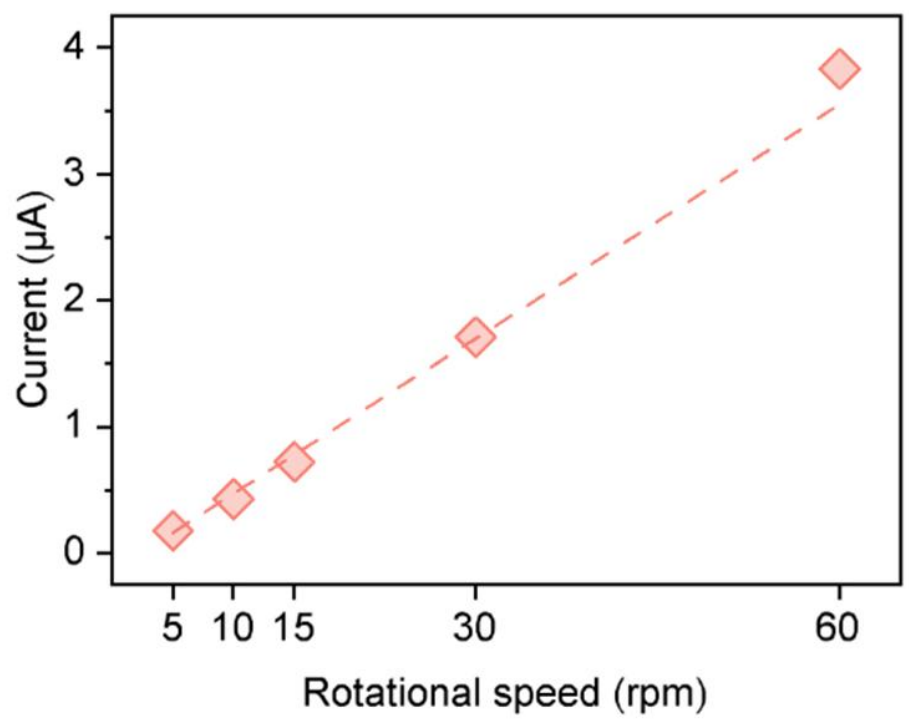

Fig. S22 | Output current amplitude of the BC-TENG as a function of rotational speed, with the corresponding data fit.

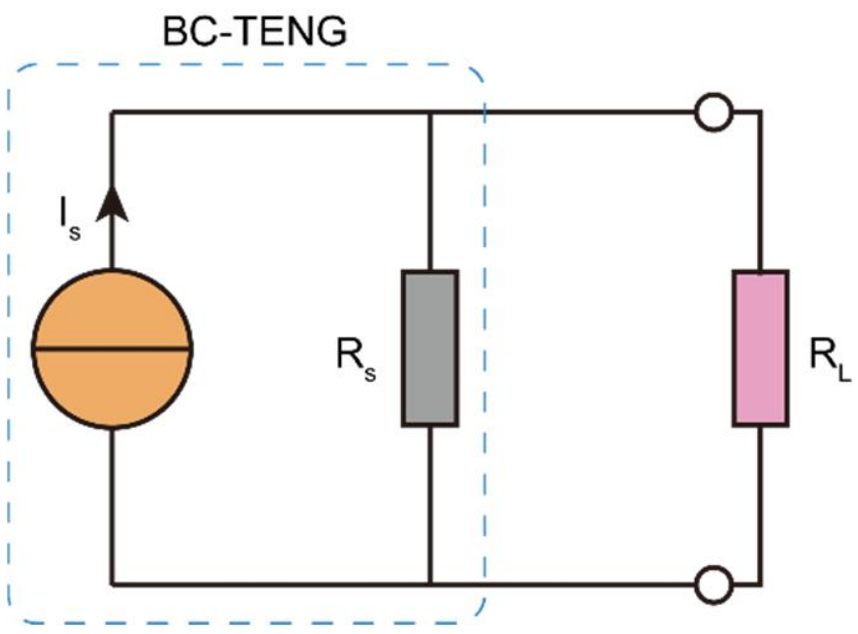

Fig. S23 | Equivalent circuit model of the BC-TENG.

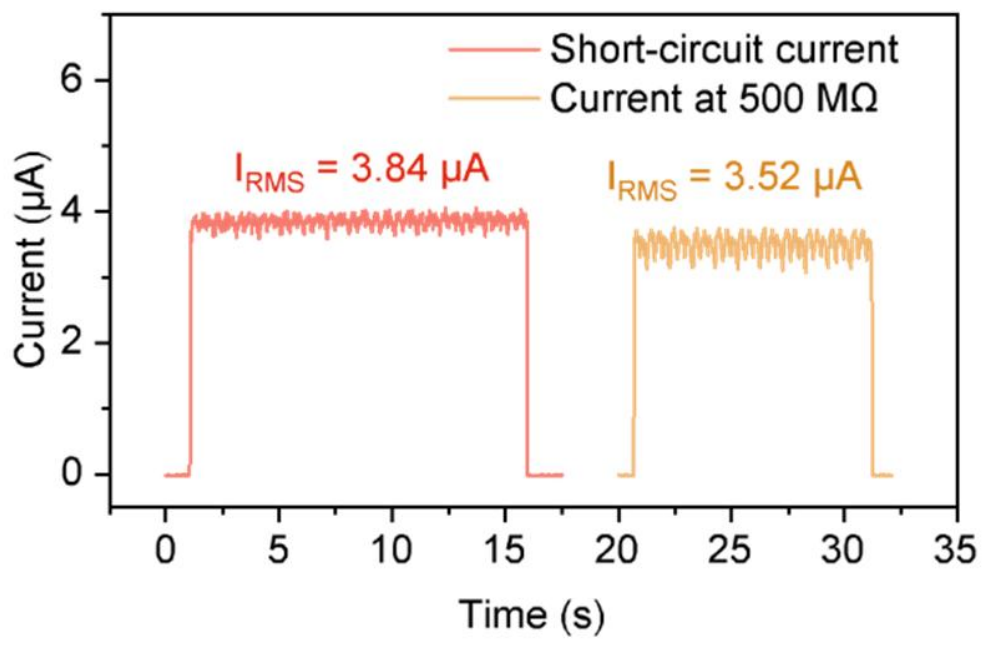

Fig. S24 | Comparison of current waveforms under short-circuit condition and across a 500 MΩ external load resistance. The root-mean-square (RMS) value of the short-circuit current was 3.84 μA. This value decreased to 3.52 μA upon connection of the 500 MΩ external load resistance, corresponding to a reduction of 8.3% (0.32 μA).

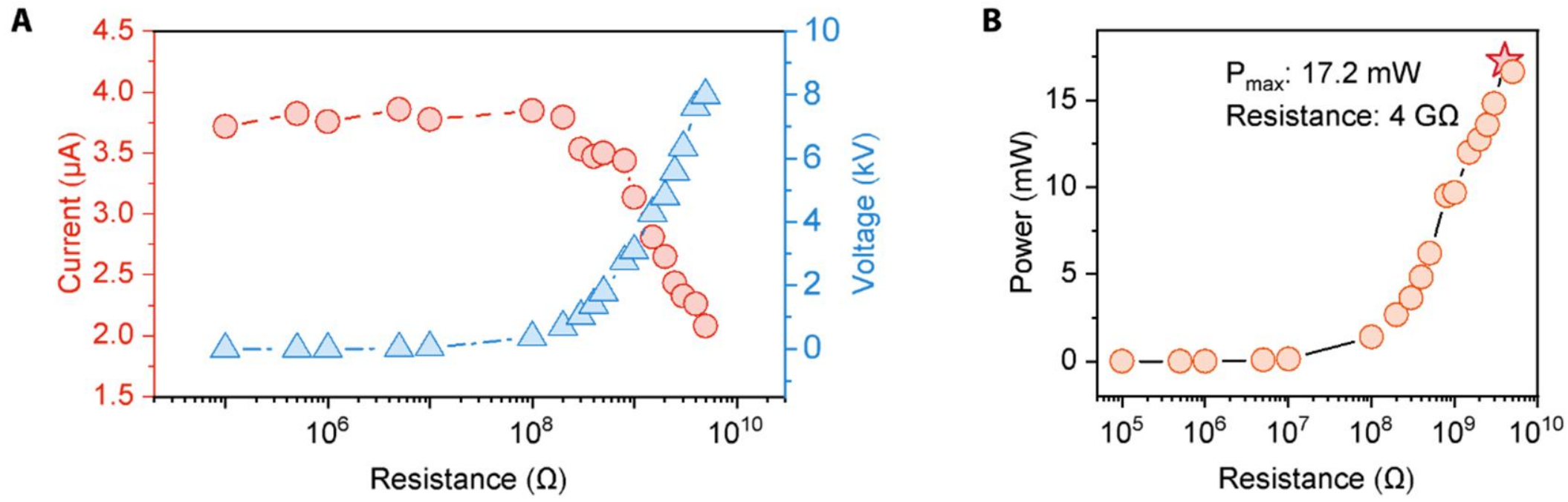

Fig. S25 | Output power of BC-TENG. (A) Output current and voltage as a function of external load resistance. (B) Output power as a function of external load resistance.

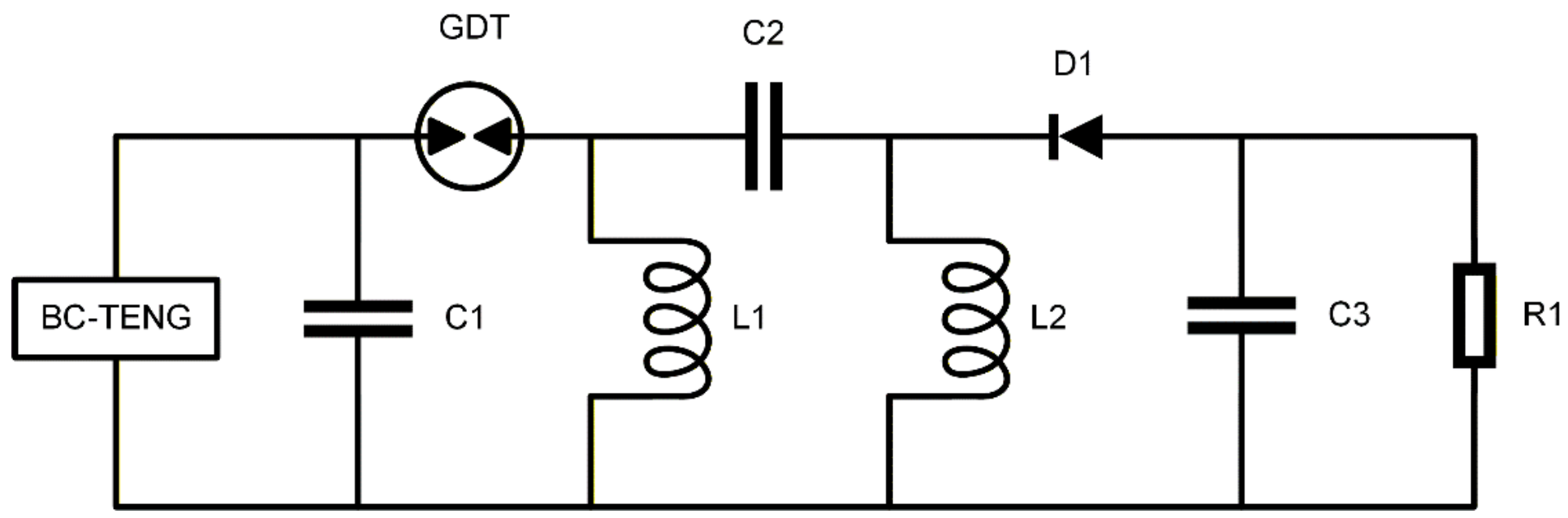

Fig. S26 | Schematic diagram of the power management circuit.

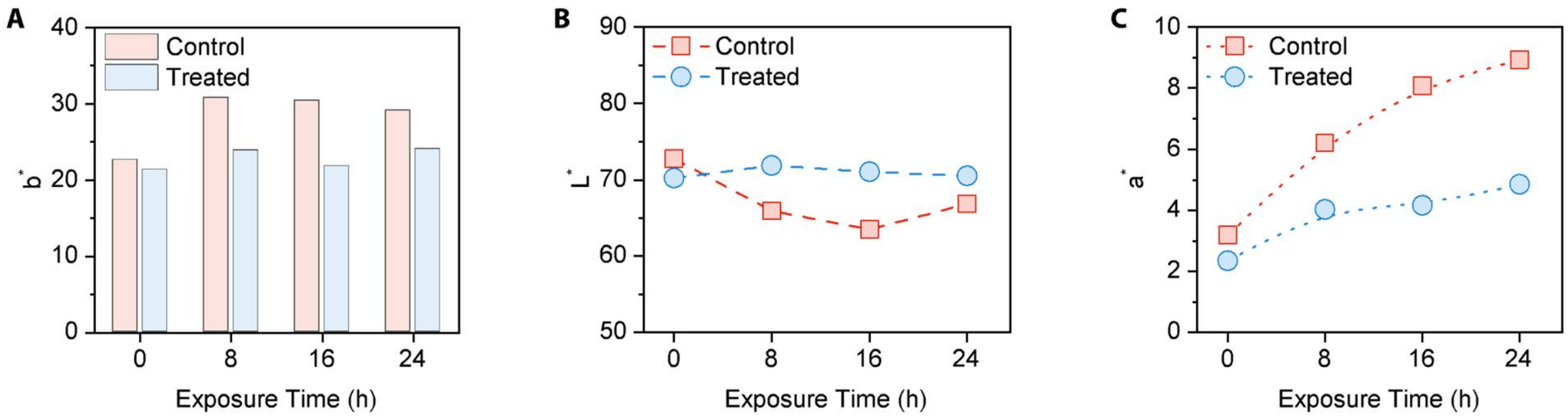

Fig. S27 | Quantitative analysis of enzymatic browning in apple slices. (A-C) Time evolution of the CIELAB color parameters b*, L, and a* for the control and treated samples.

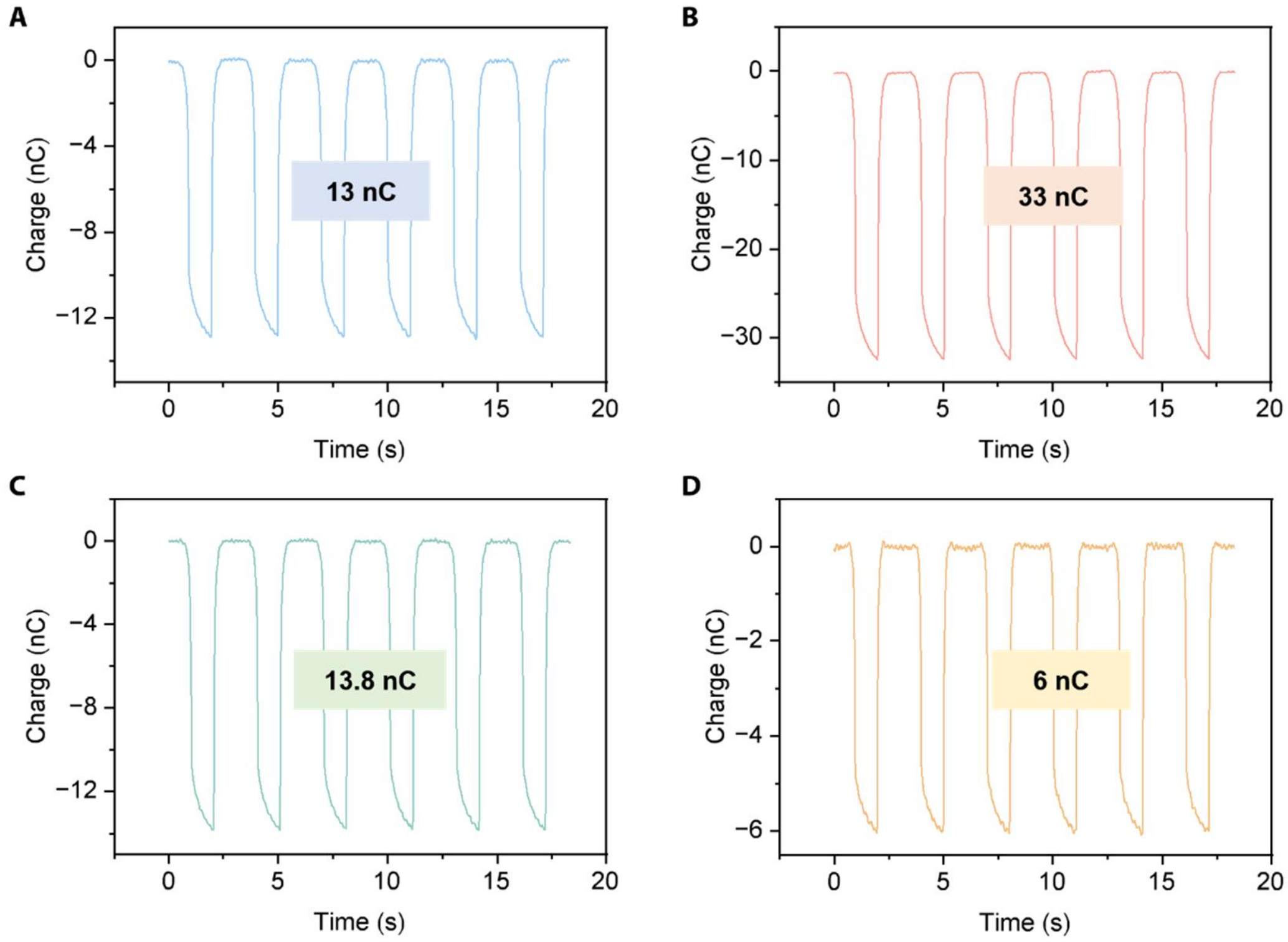

Fig. S28 | Effect of ion implantation on the transferred charge of the FEP film. (A-D Waveforms of the transferred charge measured from the FEP film under different conditions: (A) in its initial state, (B) after 50 cycles of negative ion implantation, (C) after 50 cycles of positive ion implantation, (D) after 20 cycles of positive ion implantation.

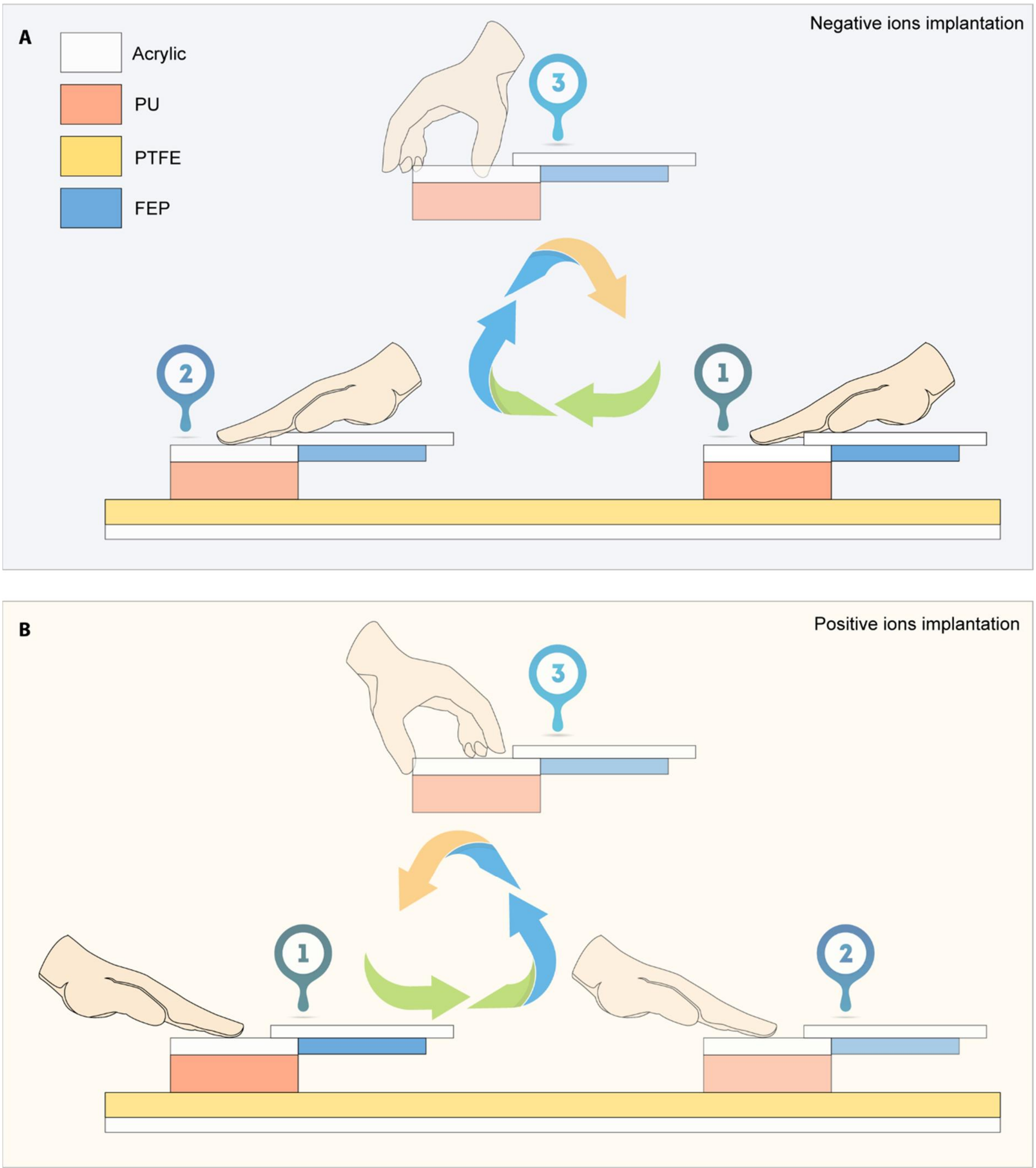

Fig. S29 | Schematic of the mechanically driven ion implantation process, showing the configurations for negative ion implantation and positive ion implantation. A 40 mm × 40 mm FEP film, adhered to an acrylic substrate of identical dimensions, was mounted on a PU slider. A gap of approximately 2 mm was maintained between the FEP surface and the opposing PTFE film. For negative ion implantation (A), the slider first contacted the PTFE film, slid backward (to the left), and then lifted off to return to its starting position. For positive ion implantation (B), the same motion cycle was performed, but with the initial sliding direction forward (to the right).

Note S1| Causes of unsaturated surface charge.

The inability of triboelectric interfacial charges to reach saturation is attributed to a continuous, dynamic "triboelectrification-discharge" microscopic cycle. This process involves two key stages. First, during initial contact, charge transfer occurs at the PU-PTFE interface owing to their different electronegativities, establishing an electric dipole layer (Fig. S1a). Crucially, the porous micro-structure of the PU surface facilitates the continuous formation of new microscopic contact regions during sliding, thereby enabling the persistent generation of triboelectric charges (the triboelectrification state). Within these nascent contact zones, the positive and negative charge centers are tightly bound at the interface, precluding discharge.

As sliding continues, however, previously contacted microscopic regions separate, creating minute gaps. This physical separation of the bound opposite charges rapidly establishes an intense and localized electric field within these gaps. Once the field strength surpasses the dielectric breakdown threshold of air, an electrostatic discharge (ESD) is triggered (the discharge state). This mechanism is further corroborated by finite element simulations, which reveal a significantly intensified electrostatic field within the gap for a charge-staggered configuration compared to a charge-aligned state (Fig. S1b). The simulations show that the field strength in these gaps can readily exceed the air breakdown threshold (~3 kV/mm), triggering discharge. These discharge events effectively neutralize the triboelectric charges within that specific microscopic region.

Consequently, at the macroscopic level, the sliding contact interface exists in a dynamic and non-uniform state, where some regions are continuously forming new contacts and accumulating charge (triboelectrification), while others are simultaneously separating and initiating discharges. It is this spatiotemporally concurrent and continuously regenerating "triboelectrification-discharge" cycle that prevents the overall surface charge density from reaching saturation.

Note S2| Current signals generated by multi-electrode array.

To elucidate the physical origin of the output signal and distinguish localized ESD from spatially extended tribo-plasma emission, we conducted a multi-electrode array experiment (Supplementary Fig. 13a). Under the localized ESD model, a discharge event would create a conductive channel, facilitating the rapid transfer and release of surface triboelectric charges. This charge release would substantially decrease the local electric field strength, thereby preventing downstream electrodes (B and C) from reaching the breakdown threshold of air. Consequently, this model predicts a current signal exclusively at the nearest electrode (A).

However, our experimental findings contradict this prediction (Supplementary Fig. 13b). The data reveal that as the PU slider advances, all three spatially separated electrodes (A, B, and C) register current signals. While the foremost electrode (A) detects the strongest current (~1 μA), distinct and non-negligible responses are concurrently observed at electrodes B and C. This multi-point detection compellingly refutes the localized ESD model. Instead, it provides direct evidence for a spatially extended phenomenon, the emission of a diffuse tribo-plasma.

Note S3| Tribo-induced emission model for tribo-plasma

To elucidate the underlying physics of the ion emission phenomenon, we develop a theoretical framework based on a simplified model of the slider's leading edge. The model considers a slider of width $\boldsymbol{w}$ moving at a constant velocity $\boldsymbol{v}$.

Over a time interval $\boldsymbol{t}$, the leading edge sweeps across a contact area $\mathbf{A_{front}(t)}$, which can be expressed as:

$$A_{\text{front}}(t) = w \cdot (vt) \qquad (1)$$

Assuming a net positive triboelectric surface charge density $\boldsymbol{\sigma_0}$, which is closely associated with intrinsic material properties (e.g., electronegativity and surface microstructure), the cumulative positive charge generated on the newly contacted area is denoted as $\mathbf{Q_{tribo}(t)}$. Accordingly, $\mathbf{Q_{tribo}(t)}$ can be expressed as:

$$Q_{\text{tribo}}(t) = \sigma_0 \cdot A_{\text{front}} = \sigma_0 wvt \qquad (2)$$

This accumulation of surface charge creates a strong local electric field that triggers the emission of positive ions from the nascent plasma via Coulombic repulsion. To quantify this process, we introduce a dimensionless ion emission efficiency $\boldsymbol{\eta}$ ($0 \leq \boldsymbol{\eta} \leq 1$), which represents the fraction of generated triboelectric charge that results in ion emission. The total number of emitted positive ions $\mathbf{N_+(t)}$, is therefore given by:

$$N_+(t) = \eta \cdot \frac{Q_{\text{tribo}}(t)}{e} = \eta \cdot \frac{\sigma_0 wvt}{e} \qquad (3)$$

The resulting ion emission current $\mathbf{I_{ion+}}$, is the time derivative of the total emitted charge ($\boldsymbol{e}\cdot\mathbf{N_+(t)}$). By differentiating Equation (3) with respect to time, we obtain a constant ion current:

$$I_{\text{ion+}} = \frac{dN_+}{dt} \cdot e = \eta \cdot \sigma_0 wv \qquad (4)$$

Note S4 | Cost analysis of rotary-mode BC-TENG

The device material cost per unit power, was calculated using, C_P, was calculated using $C_P = \frac{\sum P_i u_i}{P_{max}}$, where P_i is the commodity bulk price of each triboelectric and electrode material (PU, PTFE, and Cu), u_i is the area usage of the corresponding material, and P_{max} is the maximum output power in mW. A detailed cost calculation for the rotary-mode BC-TENG is presented in Supplementary Table S2 based on the electrical performance shown in Fig. S23. Based on these data, the calculated material cost per unit power for the BC-TENG is 0.22 \$/mW.

Table S1| Comparison of constant-current stability with representative DC-TENGs

Ref	Rotation speed (rpm)	Short-circuit current (μA)	Current at 100 MΩ (μA)	Current decay rate (μA)
Wang et al.*(38)*	300	33	~ 7	78.8%
Cheng et al.*(39)*	Sliding mode	4	~ 3.6	10%
Liu et al.*(40)*	600	17	~ 15	11.8%
Zhang et al.*(41)*	75	12.5	~6	52%
Gong et al.*(42)*	60	~79	~ 45	43%
Liu et al.*(43)*	400	39	~ 6	84.6%
Shan et al.*(44)*	/	/	~12	25%
Shan et al.*(45)*	Sliding mode	~9	~7.5	16.7%
Xu et al.*(46)*	30	40	~32	20%
**This work**	60	3.84	3.52 at **500 MΩ**	8.3%

Table S2 | Cost analysis of rotary-mode BC-TENG

Material	Quantity (Area/Mass)	Unit cost	Total cost
PTFE film	0.0119 m^2	19.89 $/m^2$	0.2367 $
Cu tape	0.0002 m^2	6.34 $/m^2$	0.0013 $
PU sheet	0.0015 m^2	23.27 $/m^2$	0.035 $
PET sheet	0.0017 m^2	2.59 $/m^2$	0.0044 $
Photopolymer resin	~135 g	0.026 $/g	3.51 $
**Total**	/	/	**3.79** $

Movie S1. BC-TENG generates electrical power by utilizing exposed or insulated wires

Movie S2. Fluctuation of positive ion concentration within a single reciprocating cycle

Movie S3. BC-TENG directly power a calculator and illuminate hundreds of LEDs

Movie S4. BC-TENG is capable of sustainably powering 20 commercial thermohygrometers

Movie S5. BC-TENG continuously powers an LED array utilizing a movable ion-collecting electrode

Movie S6. Modification of the surface wettability of FEP film via in-situ tribo-plasma treatment

REFERENCES

1. P. Ji, J. Wen, X. Gao, H. Li, H. Cai, K. Lv, Z. L. Wang, B. Chen, Roadbed tribological energy harvester. *Sci. Adv.* **11**, eadv9379 (2025).

2. D. J. Lacks, T. Shinbrot, Long-standing and unresolved issues in triboelectric charging. *Nat. Rev. Chem.* **3**, 465–476 (2019).

3. P. K. Jani, K. Yadav, M. Derkaloustian, H. Koerner, C. Dhong, S. A. Khan, L. C. Hsiao, Compressing slippery surface-assembled amphiphiles for tunable haptic energy harvesters. *Sci. Adv.* **11**, eadr4088 (2025).

4. S. Qin, X. Wang, H. Gong, X. Li, Z. Meng, L. Zheng, N. Li, Z. L. Wang, X. Chen, Ionic and adhesive triboelectric elastomer for normalizing charge polarity and density of contact electrification with general material. *Sci. Adv.* **12**, eaec1580 (2026).

5. H. Xiang, L. Peng, Q. Yang, Z. L. Wang, X. Cao, Triboelectric nanogenerator for high-entropy energy, self-powered sensors, and popular education. *Sci. Adv.* **10**, eads2291 (2024).

6. J. C. Sobarzo, F. Pertl, D. M. Balazs, T. Costanzo, M. Sauer, A. Foelske, M. Ostermann, C. M. Pichler, Y. Wang, Y. Nagata, M. Bonn, S. Waitukaitis, Spontaneous ordering of identical materials into a triboelectric series. *Nature* **638**, 664–669 (2025).

7. M. Ji, Z. Wang, J. Wu, L. Huang, M. Zheng, G. Cheng, H. Cai, J. Luo, H. Zhou, Z. L. Wang, Machine learning-assisted triboelectric nanogenerator technology for intelligent sports. *Sci. Adv.* **11**, eadz3515 (2025).

8. J. Zhang, Y. Gao, D. Liu, J.-S. Zhao, J. Wang, Discharge domains regulation and dynamic processes of direct-current triboelectric nanogenerator. *Nat. Commun.* **14**, 3218 (2023).

9. J. Wang, B. Zhang, Z. Zhao, Y. Gao, D. Liu, X. Liu, P. Yang, Z. Guo, Z. L. Wang, J. Wang, Boosting the charge density of triboelectric nanogenerator by suppressing air breakdown and dielectric charge leakage. *Adv. Energy Mater.* **14**, 2303874 (2023).

10. Y. Li, Y. Luo, S. Xiao, C. Zhang, C. Pan, F. Zeng, Z. Cui, B. Huang, J. Tang, T. Shao, X. Zhang, J. Xiong, Z. L. Wang, Visualization and standardized quantification of surface charge density for triboelectric materials. *Nat. Commun.* **15**, 6004 (2024).

11. L. He, Y. Gao, D. Liu, Y. Hu, J. Shi, J. Zhang, X. Li, B. Jin, B. Zhang, Z. L. Wang, J. Wang, Dynamic interfacial electrostatic energy harvesting via a single wire. *Sci. Adv.* **10**, eado5362 (2024).

12. Q. Zeng, A. Chen, X. Zhang, Y. Luo, L. Tan, X. Wang, A dual-functional triboelectric nanogenerator based on the comprehensive integration and synergetic utilization of triboelectrification, electrostatic induction, and electrostatic discharge to achieve alternating current/direct current convertible outputs. *Adv. Mater.* **35**, e2208139 (2023).

13. K. Nakayama, R. A. Nevshupa, Characteristics and pattern of plasma generated at sliding contact. *J. Tribol.* **125**, 780–787 (2003).

14. K. Dai, D. Liu, Y. Yin, X. Wang, J. Wang, Z. You, H. Zhang, Z. L. Wang, Transient physical modeling and comprehensive optimal design of air-breakdown direct-current triboelectric nanogenerators. *Nano Energy* **92**, 106742 (2022).

15. J. Luo, L. Xu, W. Tang, T. Jiang, F. R. Fan, Y. Pang, L. Chen, Y. Zhang, Z. L. Wang, Direct-current triboelectric nanogenerator realized by air breakdown induced ionized air channel. *Adv. Energy Mater.* **8**, 1800889 (2018).

16. Y. Gao, L. He, D. Liu, J. Zhang, L. Zhou, Z. L. Wang, J. Wang, Spontaneously established reverse electric field to enhance the performance of triboelectric nanogenerators via improving Coulombic efficiency. *Nat. Commun.* **15**, 4167 (2024).

17. Z. Zhao, Y. Dai, D. Liu, L. Zhou, S. Li, Z. L. Wang, J. Wang, Rationally patterned electrode of direct-current triboelectric nanogenerators for ultrahigh effective surface charge density. *Nat. Commun.* **11**, 6186 (2020).

18. Z. Zhao, J. Wang, Advances in interfacial electrostatic energy harvesting via direct current triboelectric nanogenerators. *Adv. Energy Mater.* **15**, 2502544 (2025).

19. S. Cui, L. Zhou, D. Liu, S. Li, L. Liu, S. Chen, Z. Zhao, W. Yuan, Z. L. Wang, J. Wang, Improving performance of triboelectric nanogenerators by dielectric enhancement effect. *Matter* **5**, 180–193 (2022).

20. C. Shan, K. Li, Y. Cheng, C. Hu, Harvesting environment mechanical energy by direct current triboelectric nanogenerators. *Nano Micro Lett.* **15**, 127 (2023).

21. D. Puhan, R. Nevshupa, J. S. S. Wong, T. Reddyhoff, Transient aspects of plasma luminescence induced by triboelectrification of polymers. *Tribol. Int.* **130**, 366–377 (2019).

22. R. T. Leon, P. C. Sherrell, J. I. Michel, J. Bullock, J. D. Berry, A. V. Ellis, Understanding and controlling electrostatic discharge in triboelectric nanogenerators. *ChemSusChem* **17**, e202400366 (2024).

23. K. Nakayama, F. Yagasaki, The flow of triboplasma. *Tribol. Lett.* **67**, 72 (2019).

24. K. Nakayama, Triboemission and triboplasma generation with DLC Films, in *Tribology of Diamond-like Carbon Films: Fundamentals and Applications* (Springer, 2007), pp. 291–310.

25. K. Nakayama, R. A. Nevshupa, Plasma generation in a gap around a sliding contact. *J. Phys. D Appl. Phys.* **35**, L53–L56 (2002).

26. K. Nakayama, Triboplasma generation and triboluminescence: influence of stationary sliding partner. *Tribol. Lett.* **37**, 215–228 (2009).

27. K. Nakayama, Triboplasma generation and triboluminescence in the inside and the front outside of the sliding contact. *Tribol. Lett.* **63**, 12 (2016).

28. R. Nevshupa, K. I. Hiratsuka, Luminescence of triboplasma: Origin, features, and behavior, in *Triboluminescence: Theory, synthesis, and application* (Springer, 2016), pp. 65–93.

29. A. von Keudell, V. S. der Gathen, Foundations of low-temperature plasma physics—An introduction. *Plasma Sources Sci. Technol.* **26**, 113001 (2017).

30. L. M. Sweeting, Triboluminescence with and without air. *Chem. Mater.* **13**, 854–870 (2001).

31. A. M. Loveless, A. L. Garner, A universal theory for gas breakdown from microscale to the classical Paschen law. *Phys. Plasmas* **24**, 113522 (2017).

32. V. Vatanpour, R. Castro-Muñoz, B. Keskin, I. Koyuncu, Plasma in fabrication and modification of polymeric membranes. *Desalination* **599**, 118444 (2025).

33. M. Bertin, E. M. Leitao, S. Bickerton, C. J. R. Verbeek, A review of polymer surface modification by cold plasmas toward bulk functionalization. *Plasma Processes Polym.* **21**, 2300208 (2024).

34. R. Alaguthevar, J. S. Packialakshmi, B. Murugesan, J. W. Rhim, U. Thiyagamoorthy, In-package cold plasma treatment to extend the shelf life of food. *Compr. Rev. Food. Sci. Food Saf.* **23**, e13318 (2024).

35. S. Kaur, Y. Kumar, V. Singh, J. Kaur, P. S. Panesar, Cold plasma technology: Reshaping food preservation and safety. *Food Control* **163**, 110537 (2024).

36. L. A. Luiz, B. C. E. S. Kurelo, G. B. de Souza, J. de Andrade, C. E. B. Marino, Effect of nitrogen plasma immersion ion implantation on the corrosion protection mechanisms of different stainless steels. *Mater. Today Commun.* **28**, 102655 (2021).

37. A. E. Illera, S. Chaple, M. T. Sanz, S. Ng, P. Lu, J. Jones, E. Carey, P. Bourke, Effect of cold plasma on polyphenol oxidase inactivation in cloudy apple juice and on the quality parameters of the juice during storage. *Food Chem. X* **3**, 100049 (2019).

38. J. Wang, H. Li, D. Zhao, Q. Gao, X. Cheng, J. Wen, Z. L. Wang, T. Cheng, High-performance and humidity-resistant direct-current triboelectric nanogenerator by coupling method combing charge excitation and phase shift. *Adv. Mater. Technol.* **8**, 2300480 (2023).

39. R. Cheng, K. Dong, P. Chen, C. Ning, X. Peng, Y. Zhang, D. Liu, Z. L. Wang, High output direct-current power fabrics based on the air breakdown effect. *Energy Environ. Sci.* **14**, 2460–2471 (2021).

40. D. Liu, X. Yin, H. Guo, L. Zhou, X. Li, C. Zhang, J. Wang, Z. L. Wang, A constant current triboelectric nanogenerator arising from electrostatic breakdown. *Sci. Adv.* **5**, eaav6437 (2019).

41. X. Zhang, D. Ren, H. Wu, J. Wang, X. Li, H. Yang, Q. Li, Q. Yang, J. Zhu, Y. Xi, Ultrahigh voltage direct current quasi-tribovoltaic nanogenerator by switchable tribo-bias induction and deposited charge extraction. *Energy Environ. Sci.* **17**, 4175–4186 (2024).

42. S. Gong, K. Li, S. Zhao, S. Gong, H. Guo, Q. Li, J. Chen, Alternating electrical polarity enabled efficient charge capture triboelectric nanogenerator for self-powered environmental purification and monitoring system. *Adv. Funct. Mater.* **36**, e11489 (2026).

43. C. Liu, X. Tong, Z. Wang, H. Lin, A. Ogunjuyibe, H. Zou, Z. Wu, W. Liu, D. Yan, B. Ma, W. Yang, L. Dong, B. Liu, G. Wang, Broadband and low-crest-factor direct-current triboelectric nanogenerator with phase-shifting modulation. *Cell Rep. Phys. Sci.* **6**, 102596 (2025).

44. C. Shan, K. Li, H. Wu, S. Fu, A. Liu, J. Wang, X. Zhang, B. Liu, X. Wang, C. Hu, Ternary electrification for efficient charge accumulation and synergetic charge collection. *Adv. Funct. Mater.* **34**, 2310332 (2024).

45. C. Shan, W. He, H. Wu, S. Fu, Q. Tang, Z. Wang, Y. Du, J. Wang, H. Guo, C. Hu, A high-performance bidirectional direct current TENG by triboelectrification of two dielectrics and local corona discharge. *Adv. Energy Mater.* **12**, 2200963 (2022).

46. S. Xu, J. Wang, C. Shan, K. Li, H. Wu, G. Li, S. Fu, Q. Zhao, Y. Xi, C. Hu, High triboelectrification and charge collection efficiency of a direct current triboelectric nanogenerator achieved by a tri-synergistic enhancement strategy. *Energy Environ. Sci.* **17**, 9590–9600 (2024).
